# Droplet-Based Radiosynthesis and High-Throughput Optimization of Vinyl Sulfone Prosthetic Group ([^18^F]FVSB) and Peptide Bioconjugation

**DOI:** 10.3390/molecules31111777

**Published:** 2026-05-22

**Authors:** Rajib K. Sarker, Jennifer M. Murphy, R. Michael van Dam

**Affiliations:** 1 Crump Institute for Molecular Imaging, University of California Los Angeles (UCLA), Los Angeles, CA 90095, USA; rsarker@mednet.ucla.edu; 2 Department of Molecular & Medical Pharmacology, University of California Los Angeles (UCLA), Los Angeles, CA 90095, USA

**Keywords:** microscale synthesis, fluorine-18 (F-18), RGDC peptide, [^18^F]FVSB-RGDC, prosthetic group, radiotracer, bioconjugation

## Abstract

Fluorine-18 is often considered an ideal positron emitter owing to its excellent chemical, physiological, and nuclear properties. Consequently, the development of rapid, simple, and reliable ^18^F-labeling strategies remains critically important for synthesizing new radiopharmaceuticals for PET molecular imaging. A common approach involves the synthesis of ^18^F-labeled prosthetic groups that subsequently undergo bioconjugation with peptides or other biomolecules to generate ^18^F-labeled imaging probes. However, conventional synthetic methods for these prosthetic groups are often lengthy, require large quantities of precursor and solvent, and typically rely on elevated reaction temperatures. Herein, we report a droplet-based microscale synthetic methodology for the preparation of the [^18^F]FVSB prosthetic group that minimizes precursor and solvent usage, proceeds rapidly, and operates at relatively low temperatures. Conditions were optimized using a platform for performing droplet reactions in parallel, enabling high-throughput study of multiple reaction parameters within a short period of time. Additionally, we introduce a simple micro-cartridge purification technique that affords purified [^18^F]FVSB in small volumes. Furthermore, we describe an efficient bioconjugation that requires substantially lower reagent amounts than the previously reported macroscale method. The microscale process we report could facilitate wider use of this ^18^F-labeling strategy and can be extended to label other thiol-bearing peptides or biomolecules.

## 1. Introduction

Positron emission tomography (PET) is a powerful nuclear imaging technique widely used to diagnose disease, monitor therapeutic response, and facilitate drug development [1]. PET relies on the annihilation of positrons with electrons, producing gamma rays that are detected by scanners to image the in vivo uptake and biodistribution of radiolabeled compounds (radiotracers) [2]. Commonly employed PET radioisotopes include carbon-11 (t_½_ = 20.4 min), fluorine-18 (t_½_ = 109.8 min), gallium-68 (t_½_ = 67.7 min), and copper-64 (t_½_ = 12.7 h) [3,4,5,6]. Fluorine-18, in particular, is often regarded as an ideal positron emitter due to its favorable chemical, physiological, and nuclear properties [7,8,9,10,11]. Accordingly, the development of rapid, simple, and reliable ^18^F-labeling strategies is essential for the synthesis of novel radiopharmaceuticals for PET molecular imaging of desired biomolecular targets.

**Figure 1:**
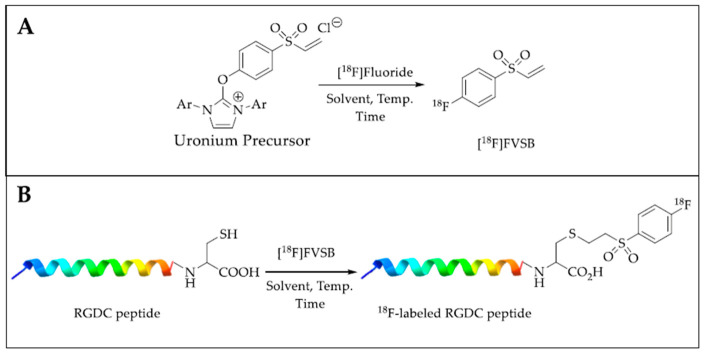
(**A**) Synthesis of thiol-reactive prosthetic group, [^18^F]FVSB via deoxyfluorination of a uronium precursor. Ar = 2,6-diisopropylphenyl. (**B**) Bioconjugation of cysteine-containing peptide with [^18^F]FVSB to afford a peptide-conjugate with a stable thioether linkage. Conditions, including reaction solvent, temperature, and time, were optimized in this work.

Radiotracers based on peptides, in particular, hold significant promise for cancer diagnosis as they have rapid in vivo kinetics, are easy to manufacture, and numerous tools exist for developing peptides that can selectively recognize protein overexpression in many cancer types [12,13,14]. In general, peptides can be radiofluorinated by two approaches: direct and indirect labeling. Direct incorporation of fluorine-18 often requires nonphysiological conditions, such as elevated temperatures or extreme pH. Most peptides and other biomolecules cannot tolerate these conditions and may undergo degradation or hydrolysis. Consequently, peptide or protein labeling is more commonly achieved indirectly using a prosthetic group. Prosthetic groups are typically conjugated to peptides via thiol-reactive or amine-reactive functionalities through reactions such as acylation, amidation, imidation, alkylation, hydrazone or oxime formation, or click chemistry. A wide variety of prosthetic groups have been developed for conjugation to peptides and small molecules, primarily using nucleophilic ^18^F-labeling strategies [15,16,17,18,19,20,21]. For instance, Ma et al. reported the synthesis and development of [^18^F]fluoro-4-(vinylsulfonyl)benzene ([^18^F]FVSB), a prosthetic group designed for site-specific conjugation to cysteine residues, yielding stable ^18^F-labeled peptides (Figure 1A) [22]. This radiosynthon is prepared in a single step via deoxyfluorination of a uronium precursor and can be employed directly without azeotropic drying or HPLC purification. [^18^F]FVSB has been used to label peptides that bind to clinically relevant receptors [23,24,25].

Despite these advances, production methods relying on conventional commercial radiosynthesizers face several limitations, including high equipment and operating costs, lengthy synthesis times, and restricted synthesis capacity per day. Furthermore, to maintain reasonable reaction rates at the milliliter scale, large amounts of precursors and reagents are required, leading to challenges in downstream purification. To address these limitations, recent studies, including work from our group and others, have shown that microscale synthesis methods enable efficient reactions in a compact setup with dramatically reduced reagent quantity and synthesis time [26,27]. Smaller reagent quantities lead to reduced amounts and types of impurities, facilitating the use of rapid purification methods (e.g., microscale solid-phase extraction (SPE) or analytical-scale HPLC) for radiopharmaceuticals produced at the microscale.

Microfluidic technologies have gained attention as compact, efficient, and economical platforms with significant potential for radiotracer synthesis [26,28]. Broadly, these systems fall into two categories: continuous-flow reactors and microscale batch reactors. Although flow-based devices can support the production of many radiopharmaceuticals, they often remain comparable to conventional synthesizers in terms of overall size, shielding needs, and reaction volumes [29]. In contrast, batch-based microsystems can deliver clinically relevant quantities of radiotracers while offering clear advantages, including reduced reagent consumption, smaller footprint, and straightforward integration with upstream and downstream operations. Several configurations have been developed, such as microvial reactors [30], channel-based architectures [31], and droplet-based platforms [27,32]. Our work has focused on droplet-based formats due to their simplicity, fast reaction cycles, and versatility. Moreover, this approach enables larger-scale production—such as single or multiple clinical doses—under identical reaction conditions by simply increasing the starting activity when using an automated droplet-based radiosynthesizer [33,34]. Reactions take place on silicon “chips” that have been coated with Teflon AF, and circular patches of the coating are etched away to expose the silicon surface. Droplets placed on these patches are held in place by surface tension. In addition to single-reaction devices for PET tracer production, a multi-reaction droplet array chip has been developed that enables simultaneous execution of multiple reactions at the 1–20 μL scale for optimization purposes [35]. Operating multiple chips in parallel enables high-throughput exploration of reaction conditions, including variation in reagent concentrations, solvents, reaction temperature, and time, all while consuming minimal amounts of precursor.

In this study, we apply the microscale droplet reaction methods to optimize and improve the preparation of the versatile prosthetic group [^18^F]FVSB (Figure 1A) and its subsequent conjugation to an RGDC peptide (Figure 1B). Our objectives were to reduce reagent consumption, accelerate the overall synthetic process, and conduct reactions under milder conditions with improved yields. We leveraged a workflow of first optimizing each reaction step using the high-throughput platform. Subsequently, we showed that the optimized conditions could be scaled to higher activity levels, sufficient for preclinical *in vivo* imaging. Furthermore, as part of this project, we show the possibility of performing two-pot reactions in microscale droplet processes for the first time—a new capability for microscale droplet radiochemistry. Prior work in droplet radiochemistry has focused only on one-pot reactions [27,32].

## 2. Results

### 2.1. Optimization of Microscale [^18^F]FVSB Synthesis

#### 2.1.1. Overview

**Figure 2:**
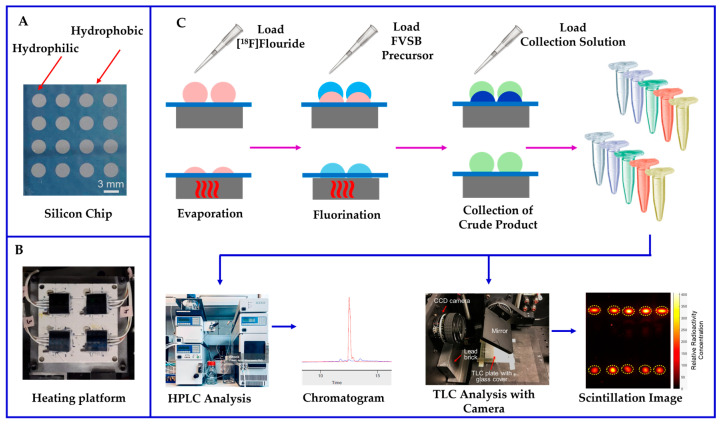
(**A**) Multiple reaction chip with a 4 × 4 array of reaction sites for high-throughput synthesis optimization. (**B**) Heating platform with four chips installed. (**C**) Workflow of droplet-based radiosynthesis of [^18^F]FVSB prosthetic group on multiple reaction sites in parallel. After addition and drying of [^18^F]fluoride solution, followed by addition and reaction with FVSB precursor, the crude products are collected from the chip and analyzed via multi-lane radio-TLC or radio-HPLC. Reactions at different sites can be performed under different conditions for optimization purposes.

We initially translated the conventional vial-based synthesis of the [^18^F]FVSB prosthetic group to microscale and optimized the conditions using high-throughput droplet radiochemistry techniques (Figure 2). Briefly, the synthesis begins by combining [^18^F]fluoride, a base, and a phase transfer catalyst (PTC) and removing water by evaporation to form an activated [^18^F]fluoride complex. Next, a solution of FVSB precursor is added, and the resulting mixture is heated to perform the radiofluorination reaction. Finally, crude products are collected from the chip, with the aid of multiple rinses using a collection solution. To characterize the performance of each microscale reaction, we evaluated the radiochemical conversion (RCC; determined via radio-TLC analysis of the collected crude product), the collection efficiency (Coll. Eff.; determined by dividing radioactivity of the collected crude product by the initially loaded [^18^F]fluoride radioactivity), and the crude radiochemical yield (crude RCY; determined by multiplying RCC and collection efficiency).

#### 2.1.2. Initial Conditions

As a starting point for the droplet-based synthesis of [^18^F]FVSB (Figure 2), we scaled down the reaction volume used in Ma et al. [22] from 1 mL to 10 µL (100-fold reduction) and also proportionately scaled down the amount of base used in the [^18^F]fluoride drying process (from 5 mg to 0.05 mg). However, we modified some parameters based on more recent developments in the macroscale synthesis. For example, Hu et al. and Wang et al. conducted the labeling reaction at 90 °C [23,24] which we adopted as an initial temperature for the droplet reaction. We selected a reaction time of 1 min since we have found other droplet-based reactions to proceed this quickly, e.g., our recent report on the droplet-based radiosynthesis of [^18^F]fluorobenzyltriphenylphosphonium ([^18^F]FBnTP) [33]. In addition, based on our previous work demonstrating that DMA is a suitable solvent for the radiosynthesis of various radiotracers, DMA was selected as the initial reaction solvent [35]. Also, for a previous deoxyfluorination radiolabeling of a similar uronium precursor, Narayanan et al. reported TEAHCO_3_ as the base used for the synthesis of 4-[^18^F]fluorophenyl sydnone [36]; therefore, we adopted TEAHCO_3_ as our initial base prior to optimization.

#### 2.1.3. Influence of Precursor Amount

**Figure 3:**
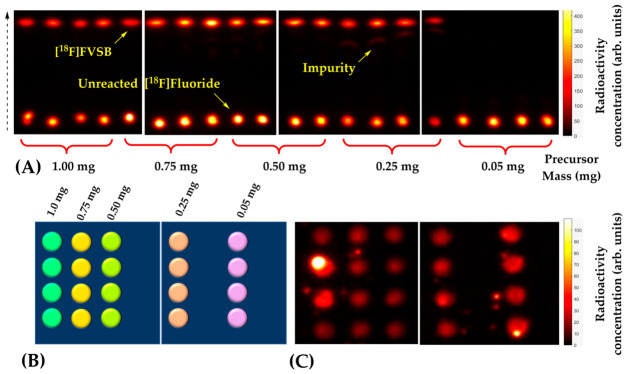
Experimental conditions and results for the study of precursor amount. (**A**) Scintillation images of developed TLC plates showing bands for unreacted [^18^F]fluoride, [^18^F]FVSB, and a minor impurity. Dashed arrow represents propagation of mobile phase. (**B**) Map of reaction conditions on two multi-reaction chips showing the amount of precursor used in each microscale reaction. There are n = 4 replicates of each condition. (**C**) Scintillation images of residual activity at reaction sites after collecting the crude products.

**Figure 4:**
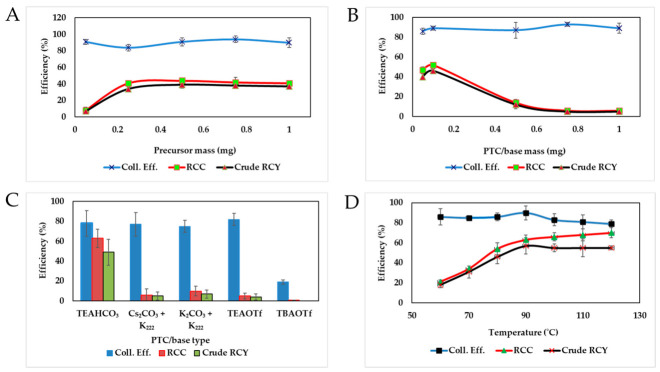
Influence of reaction parameters on the performance of the microdroplet radiosynthesis of [^18^F]FVSB. (**A**) Impact of precursor mass. (**B**) Impact of PTC/base mass. (**C**) Impact of PTC/base type. (**D**) Impact of temperature. Each condition was repeated n = 4 times, with a few exceptions where n = 3. Error bars represent the standard deviation.

To assess the influence of precursor amount on the reaction performance, reactions were performed with 0.05 mg (0.26 µmol) of TEAHCO_3_ during the F-18 drying step. Varying precursor amounts (dissolved in 10 μL of DMA) were added, and the reaction was heated to 90 °C for 1 min to perform the fluorination. Detailed measurements and calculations are provided in Table S1, while scintillation images of radio-TLC plates and residual activity on reaction chips are shown in Figure 3. The precursor amount had a significant impact on performance (Figure 4A). Increasing precursor mass improved the RCC and collection efficiency, reaching maximum values using ~0.5 mg of precursor, obtaining a RCC of 44 ± 2% (n = 4), a collection efficiency of 91 ± 5% (n = 4), and a crude RCY of 39 ± 4% (n = 4). At higher precursor masses, there are slight decreases in RCC and hence crude RCY. Therefore, 0.5 mg was selected as the optimal precursor mass.

#### 2.1.4. Effect of PTC/Base Amount

The effect of the PTC/base amount was investigated next. All reactions were performed with the desired amount of TEAHCO_3_ as the PTC/base during the F-18 drying step. Precursor (0.5 mg, 0.82 µmol) dissolved in 10 μL of DMA was added, and the reaction was heated to 90 °C for 1 min. Detailed measurements, calculations, and scintillation images of radio-TLC plates and residual activity on chips are provided in Table S2 and Figure S3. The results are summarized in Figure 4B. The PTC/base mass has a strong impact on RCC. With increasing PTC/base amount, both RCC and crude RCY improved, reaching maximum values at 0.1 mg, with RCC of 52 ± 2% (n = 4), collection efficiency of 89 ± 2%, and crude RCY of 46 ± 2% (n = 3). However, when the base mass exceeded 0.1 mg per reaction site, the performance abruptly decreased. Based on these findings, 0.1 mg of PTC/base was selected as optimal.

#### 2.1.5. Influence of PTC/Base Type

Based on a literature survey, we selected several commonly used compositions for the PTC/base: Cs_2_CO_3_/K_222_, K_2_CO_3_/K_222_, TEAHCO_3_, TEAOTf, and TBAOTf [37,38,39]. Microscale reactions were performed with 0.1 mg of each of these PTC/base combinations during the F-18 drying step; subsequently, 0.5 mg (0.82 µmol) of precursor in 10 μL of DMA was added and heated at 90 °C for 1 min. Reactions involving Cs_2_CO_3_ and K_2_CO_3_ were conducted with K_222_ (0.083 mg, 0.297 mmol) as the PTC. Detailed measurements and calculations are provided in Table S3, while multi-lane TLC data and residual activity distribution on chips are presented in Figure S4. The results are summarized in Figure 4C. Among the bases tested, TEAHCO_3_ demonstrated the highest fluorination conversion of 63 ± 9% (n = 4) and the highest crude RCY of 49 ± 13% (n = 4) with a collection efficiency of 78 ± 13% (n = 4). For Cs_2_CO_3_/K_222_, K_2_CO_3_/K_222_, and TEAOTf, the collection efficiency was high (77–82%), but radiochemical conversion was much lower (5–10%), resulting in very low crude RCY values (4–7%). TBAOTf exhibited almost no fluorination and had a low collection efficiency of only 19 ± 2% (n = 4). Based on these findings, TEAHCO_3_ was selected as the optimal base for the microscale preparation of [^18^F]FVSB.

#### 2.1.6. Influence of Reaction Solvent

Literature precedents indicate that DMI, DMA, DMSO, MeCN, 2-butanone, and EtOH, or combinations, are suitable solvents for radiofluorination [40,41]. To compare these solvents, microscale reactions were performed using TEAHCO_3_ (0.1 mg, 0.52 μmol) during the F-18 drying step and 0.5 mg (0.82 μmol) of precursor in 10 μL of the desired solvent at 90 °C for 1 min for the fluorination reaction. Detailed measurements and calculations are provided in Table S4, and scintillation images of the multi-lane TLC plates and residual activity on chips are shown in Figure S5. The results are summarized in Figure S6. Among the solvents tested, DMA exhibited the highest performance, achieving a fluorination conversion of 59 ± 5% (n = 4), a collection efficiency of 86 ± 5% (n = 4), and a crude RCY of 52 ± 5% (n = 4). DMF also emerged as a viable alternative, demonstrating performance comparable to that of DMA. Other solvent systems tested included DMI + pyridine (24:1, *v*/*v*), MeCN + DMSO (3:1, *v*/*v*), and 2-butanone + EtOH (10:1, *v*/*v*), which afforded significantly lower crude RCYs of 24%, 20%, and 44%, respectively. The exact compositions of these solvent mixtures were selected based on previously published reports demonstrating their suitability for radiofluorination.

#### 2.1.7. Effect of Temperature

During subsequent investigation of the effect of reaction temperature, the following parameters were kept constant: TEAHCO_3_ as the base (0.1 mg, 0.52 μmol), FVSB precursor (0.5 mg, 0.82 μmol), a reaction time of 1 min, and DMA as the reaction solvent. Comprehensive measurements, calculations, and scintillation images of radio-TLC plates and residual activity on chips are provided in Table S5 and Figure S7. Results are summarized in Figure 4D. The RCC increased significantly with temperature, reaching a maximum of 70 ± 5% (n = 4) at 120 °C. Collection efficiency was relatively constant, with a slight decrease above 90 °C. The crude RCY increased sharply with temperature, rising to a maximum value at 90 °C, and then leveling off beyond 90 °C. Temperatures above 90 °C were found to induce impurity formation, as evidenced by radio-TLC measurements (Figure S7). This could be analogous to the radioactive impurity we observed in the deoxyfluorination process for another compound [21]. The highest crude RCY (57 ± 8%, n = 4) was obtained at 90 °C, corresponding to a fluorination conversion of 63 ± 5% (n = 3) and a collection efficiency of 90 ± 7% (n = 4).

#### 2.1.8. Influence of Reaction Time

We next assessed the impact of reaction time. For all experiments, the following factors were kept constant: TEAHCO_3_ as the base (0.1 mg, 0.52 μmol), FVSB precursor (0.5 mg, 0.82 μmol), a reaction temperature of 90 °C, and DMA as the reaction solvent. Detailed measurements, calculations, and scintillation images of radio-TLC analysis are provided in Table S6 and Figure S8. The results are summarized in Figure S9. The reaction time had a minimal influence on fluorination performance. RCC, collection efficiency, and crude RCY remained consistent, except when the reaction time exceeded 2 min, leading to the formation of impurities. Since a satisfactory crude RCY was achieved within 1 min and the reaction remained relatively clean, 1 min was selected as the optimal reaction time.

#### 2.1.9. Influence of Collection Solvent

To assess the effect of the collection solvent, reactions were performed under the optimal conditions, namely 0.1 mg of TEAHCO_3_ during the drying step, and 0.5 mg of the precursor in DMA at 90 °C for 1 min. Crude products were collected using different solutions. Detailed measurements, calculations, and the scintillation images of the radio-TLC plates and residual activity on the chip are provided in Table S7 and Figure S10. The results are summarized in Figure S11. The collection efficiencies were high for most of the solvent mixtures used in this experiment. Among the six tested options, a 1:1 (*v*/*v*) mixture of MeCN and H_2_O was identified as the most suitable collection solvent.

### 2.2. Optimization Results of Microscale ^18^F-Labeling of an RGDC Peptide

#### 2.2.1. Initial Conditions

We first synthesized [^18^F]FVSB in microscale under the optimal conditions described above. Optimization of the bioconjugation reaction was performed starting with the crude [^18^F]FVSB droplet without an intermediate purification step. For the initial conditions, RGDC peptide (0.5 mg) in a mixture of sodium borate buffer (pH 8.5) and MeOH (1:1, *v*/*v*) was added to the reaction site (already containing crude [^18^F]FVSB), followed by heating at 35 °C for 5 min. The buffer composition was selected from the previously published Ma et. al. report [22]. HPLC analysis showed no detectable product under these conditions. We then substituted the borate buffer with HEPES buffer (pH 7.3) while keeping all other parameters unchanged. In this case, HPLC revealed the formation of a small amount of the ^18^F-labeled peptide, and therefore, HEPES buffer (pH 7.3) was selected for subsequent optimization studies.

#### 2.2.2. Optimization of Bioconjugation Time

The effect of reaction time on the bioconjugation process was evaluated next. The prosthetic group was first synthesized on a silicon chip using the optimized conditions. Following [^18^F]FVSB synthesis, a 10 μL solution of the RGDC peptide (0.5 mg, 1.11 µmol) prepared in a mixture of methanol and HEPES buffer (pH 7.3) (1:1, *v*/*v*) was added to the crude [^18^F]FVSB, and the bioconjugation was performed for varying lengths of time at 37 °C. Detailed measurements and calculations, together with scintillation images of radio-TLC and residual chip activity, are provided in Table S8 and Figure S12, respectively. Results are summarized in Figure S13. The collection efficiency was relatively constant, and the bioconjugation efficiency increased gradually with reaction time. A reaction time of 10 min was selected as optimal for bioconjugation.

#### 2.2.3. Optimization of Bioconjugation Temperature

The effect of temperature on bioconjugation was investigated similarly. A 10 µL aliquot of RGDC peptide in a mixture of MeOH and HEPES buffer (pH 7.3) (1:1, *v*/*v*) was added to the crude [^18^F]FVSB at each reaction site, and bioconjugation was performed for 10 min at different temperatures to analyze its effect. Detailed measurements and calculations, along with scintillation images of radio-TLC and residual chip activity, are presented in Table S9 and Figure S14, respectively. The results are summarized in Figure S15. It is evident that the temperature has little influence on bioconjugation, but the highest performance was observed at 55 °C, where RCC was 24 ± 1%, collection efficiency was 86 ± 2%, and crude RCY was 21 ± 1%.

### 2.3. Overall Synthesis of F-18 Labeled RGDC Peptide in a One-Pot Process

**Figure 5:**
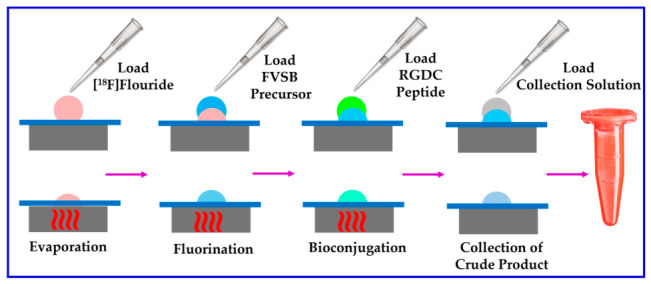
Workflow for microscale radiosynthesis of ^18^F-labeled peptide via a one-pot approach. First, [^18^F]FVSB is synthesized on a silicon chip at the desired activity scale via [^18^F]fluoride drying and radiofluorination of the FVSB precursor. Second, the reagents for the bioconjugation reaction are added to the reaction sites, and the bioconjugation is performed. Finally, the crude products are collected.

After optimization of the prosthetic group synthesis and bioconjugation conditions, we assessed the overall performance of this one-pot microscale labeling strategy (Figure 5). As described in Section 2.1, the optimal conditions for the synthesis of [^18^F]FVSB used 0.1 mg TEAHCO_3_ during drying of [^18^F]fluoride, 0.5 mg FVSB precursor in 10 μL DMA, heated to 90 °C for 1 min. Furthermore, the optimal conditions for the bioconjugation step (Section 2.2) were identified as 0.5 mg RGDC peptide in 10 μL of a methanol–HEPES buffer mixture (pH 7.3, 1:1 *v*/*v*), heated to 55 °C for 10 min. Upon completion, the final ^18^F-labeled RGDC peptide was collected and analyzed by scintillation imaging.

This one-pot synthesis afforded an overall collection efficiency of 83 ± 3%, a radiochemical conversion of 26 ± 2%, and a crude radiochemical yield of 21 ± 2% (Table S10). The overall synthesis time from loading [^18^F]fluoride to collecting the final crude ^18^F-labeled peptide was 22 min.

### 2.4. Exploration of Two-Pot Microscale Synthesis

We hypothesized that the bioconjugation reaction performance may be hindered by the presence of a significant amount of unreacted FVSB precursor, which would compete with the radiolabeled prosthetic group for conjugation with the RGDC peptide, thereby reducing the RCC. We explored the integration of an intermediate purification of [^18^F]FVSB as a potential strategy to improve bioconjugation efficiency to achieve higher overall RCY. For the synthesis of the ^18^F-labeled peptide, we first prepared [^18^F]FVSB under the previously optimized conditions. After synthesis, [^18^F]FVSB was purified using a custom-made micro-cartridge packed with 13 mg of HLB resin. The cartridge fabrication method and optimization of the purification conditions were adapted from Lu et al. and are described in the Supplementary Materials (Section S4.4) [42]. Briefly, the crude product was diluted 10× with DI water, followed by a trapping step on the cartridge, and then elution of the pure [^18^F]FVSB using 120 µL of MeOH. The eluted [^18^F]FVSB was transferred onto two new reaction sites on the microdroplet reaction chip, with each site receiving three sequentially loaded aliquots (20 µL) with a solvent evaporation step after each aliquot was delivered. (We found that if the entire amount of purified [^18^F]FVSB were to be loaded onto a single reaction site, there could be a loss of [^18^F]FVSB via decomposition or evaporation due to the prolonged time needed to load and evaporate 6 sequential aliquots.) An additional 40 µL of MeOH was used to rinse residual [^18^F]FVSB from the vial and was divided among the two reaction sites. Each evaporation step was performed by heating at 65 °C for 30 s, followed by an additional heating at 70 °C for 30 s. The bioconjugation reaction was performed under the previously optimized conditions: RGDC peptide solution (0.5 mg peptide in 10 µL of a mixture of MeOH and HEPES buffer (pH 7.3) (1:1, *v*/*v*) was added to each of the two reaction sites, and the chip was heated at 55 °C for 10 min. The final ^18^F-labeled peptide was collected from each of the two reaction sites using 60 µL of MeCN and water (1:1, *v*/*v*). The final products from the two sites were combined and analyzed further through radio-TLC and HPLC. Remarkably, the two-pot strategy greatly improved the performance, resulting in a crude RCY of 37 ± 3% for the overall synthesis. Even though including the purification step lengthened the synthesis time (by 24 min), which leads to around 14% decay, the crude activity yield remains significantly higher for the two-pot process (28 ± 3%) compared to the one-pot process (18 ± 2%), i.e., >50% more product.

## 3. Discussion

**Figure 6:**
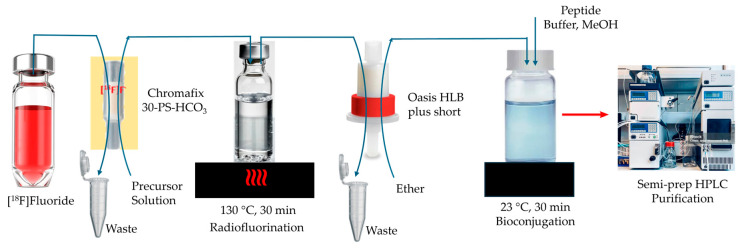
The conventional method of performing peptide labeling, including synthesis and purification of [^18^F]FVSB, followed by bioconjugation and semi-preparative HPLC purification.

Our previous report described the conventional (macroscale) synthesis of [^18^F]FVSB and its bioconjugation with various peptides [22]. Compared to the microscale process, the vial-based process (Figure 6) requires much larger amounts of precursors and other reagents, larger solvent volumes, and extended reaction times. Furthermore, optimization of the vial-based procedure was time-consuming since only a small number of reactions could be performed per day. The vial-based synthesis of the prosthetic group used 5 mg of the FVSB precursor dissolved in 1 mL of a butanone/EtOH mixture (10:1, *v*/*v*), and the fluorination reaction was carried out at 130 °C for 30 min. The RCC (determined via HPLC) was 94 ± 7%, and the (isolated) RCY was 46 ± 4%, corresponding to an activity yield of 32 ± 3% based on an [^18^F]FVSB synthesis time of 56 min [22]. (Crude RCY was not reported.) A review of the literature reveals a few other precedents for [^18^F]FVSB syntheses (Table 1). Hu et al. reported a synthesis with RCY of 41% (determined via HPLC) [23]. Craig et al. reported a synthesis with RCC of 83 ± 2% (determined via UHPLC) and an activity yield of 18 ± 3%, calculated as the ratio of (isolated) product activity to initial starting activity [43]. These studies used precursor amounts ranging from 2.0 to 5 mg, temperatures ranging from 90 to 130 °C, and reaction times from 20 to 30 min.

The present microscale study shows that [^18^F]FVSB can be synthesized using only 0.5 mg of precursor and 0.1 mg of base in a 10 µL droplet reaction, at 90 °C with a reaction time of only 1 min. This is a significant reduction in reagent amount and time compared to the conventional vial-based reaction. The droplet-based method generates the radiosynthon using 4–10-fold less precursor, 8–120-fold less solvent, and the fluorination reaction is approximately 20–30× faster. Furthermore, the microscale reaction exhibited high performance. The RCC (determined via TLC) was 74 ± 2%, with crude RCY of 64 ± 2%. In our study, the isolated RCY of [^18^F]FVSB was 59 ± 3% (n = 4), which corresponds to 51 ± 3% activity yield. The combined microscale synthesis and purification time was 22 min. The molar activity at the end of synthesis (EOS) was determined to be >45 GBq/μmol. Table 1 compares the conditions and performance of the present microscale study with vial-based reactions reported in the literature.

For the microscale purification of [^18^F]FVSB (using 13 mg of HLB resin), we achieved 70 ± 3% trapping efficiency (10 min trapping time) and 98 ± 2% elution efficiency (2 min elution time), recovering the purified product in 120 µL of eluent with 69 ± 3% efficiency (Table S11). In our previously reported macroscale synthesis, purification was performed using a commercial HLB cartridge (Oasis, 250 mg) [22]. The trapping efficiency was 88%, and the product was eluted using 2 mL of ether with 79% elution efficiency, corresponding to an overall purification efficiency of 70%. Although the overall performance is comparable, the microscale purification method developed in this study requires 19-fold less resin and 16-fold less elution solvent.

Looking at the bioconjugation reaction, we previously reported a vial-based process that required 3 mg of peptide, 500 µL solvent, and a reaction time of 30 min [22]. The RCC (conversion of initial [^18^F]FVSB to conjugated peptide; determined via HPLC) was 89 ± 6%, and the crude RCY (factoring in the amount of radioactivity recovered at the end of the reaction) was 84 ± 8% (relative to purified [^18^F]FVSB and only for the bioconjugation step). The literature study reveals a few additional precedents of biomolecule conjugation with [^18^F]FVSB (Table 1), though not with RGDC peptide. Hu et al. reported bioconjugation between amino acid derivatives and [^18^F]FVSB, observing high RCCs of 97–99% (determined via HPLC) and crude RCYs of 42–60% [23]. Wang et al. described the bioconjugation of a PSMA precursor using several prosthetic groups, including [^18^F]FVSB. Though the reaction performance for [^18^F]FVSB conjugation in particular was not reported, the authors indicated that the conjugation reactions had crude RCYs ranging from 18 ± 1% to 53 ± 3% (presumably relative to the starting amount of the prosthetic group) [24]. Craig et al. reported bioconjugation between [^18^F]FVSB and cysteine-containing compounds, yielding RCCs of 98–99% [43].

For the microscale process, bioconjugation with RGDC peptide was carried out in a 1:1 (*v*/*v*) mixture of MeOH and HEPES buffer (pH 7.3). The bioconjugation reaction required only 0.5 mg × 2 (1.11 µmol × 2) of peptide in 10 µL × 2 of solvent and was completed in 10 min at 55 °C. Comparing the RGDC peptide conjugation, the droplet-based method used 3-fold less precursor, 25-fold less solvent, and the bioconjugation reaction time was three times faster than our previously reported macroscale synthesis. For the microscale bioconjugation reaction starting from purified [^18^F]FVSB, we observed an RCC of 74 ± 4% and crude RCY of 55 ± 4%. Considering the entire process from initial [^18^F]fluoride activity loaded on the chip to the ^18^F-labeled RGDC conjugate, the overall crude RCY was 37 ± 3%. The overall synthesis time was 46 min. The apparent molar activity at EOS of the ^18^F-labeled peptide was determined to be 0.588 GBq/μmol. Table 1 compares the conditions and performance of the peptide bioconjugation with [^18^F]FVSB discussed in this work versus those reported in the literature.

The findings in this work also emphasize the effectiveness of droplet-based platforms, enabling high-throughput capabilities to perform reaction optimization while using only minimal reagent quantities. An additional advantage of this microdroplet setup is the ability to routinely visualize and quantify radioactivity on the chip surface through Cerenkov or scintillation imaging, for example, to quantify the amount of residual activity left behind on the chip. This enables routine and thorough accounting of the droplet reaction performance, not only measuring the RCC, but also the losses, which result in a lower crude RCY. It is evident in some reports of the conventional synthesis that activity losses can also be very significant in vial-based reactions, i.e., some reports have a large discrepancy between RCC and RCY values. However, unless painstaking efforts are made to measure residual activity in the reaction vessel(s), tubing, and other fluidic pathways, it can be difficult to identify and reduce such losses. One potential limitation of droplet reactions is the possibility for volatile losses during certain syntheses, as the open droplet geometry can allow evaporation during reaction steps. By comparison, vial-based systems typically experience lower volatile losses because the vial remains sealed during reactions, though they can also experience losses during solvent evaporation steps. Nevertheless, even with some volatilization, the droplet platform enabled experiments that were reproducible, scientifically meaningful, and resulted in improved reaction performance.

In addition to improving the synthesis of [^18^F]FVSB and subsequent bioconjugation reactions, this microscale work also expands the range of synthesis processes performed in droplet reactions. In particular, this study is the first example of performing a two-pot droplet microreaction, namely, an initial synthesis of [^18^F]FVSB in a droplet reaction, followed by off-chip microscale SPE purification and reloading to the chip, followed by a second microscale droplet reaction (bioconjugation). Previous examples of droplet-based radiochemistry have focused only on 1-pot reactions where no intermediate purification is performed between multiple reactions.

Building upon this successful microscale optimization, future studies can extend the bioconjugation of [^18^F]FVSB to other peptides already established as radiotracers. Additional efforts should also focus on minimizing activity loss during purification and reloading of [^18^F]FVSB onto the chip to further improve RCY. Droplet reactions can be scaled up to amounts sufficient for one or multiple clinical doses by preconcentrating the [^18^F]fluoride using microscale-cartridge-based methods [44], repeated activity loading [34], or using multiple reaction sites [33]. Ultimately, automation of the entire workflow would not only improve efficiency but also minimize radiation exposure to personnel. Our previously developed automated droplet-based radiosynthesizer [45] that is currently undergoing development and integration with a compact HPLC system would be suitable for this purpose. Extension of that platform to include an automated mechanism to perform the microscale intermediate purification would further increase the versatility of automated droplet-scale radiochemistry. A commercial prototype of the microscale technology is currently in development by DropletPharma Corp. (Los Angeles, CA, USA), and is expected to be fully capable of supporting cGMP production of radiotracers.

## 4. Materials and Methods

### 4.1. General Reagents and Materials

Tetraethylammonium trifluoromethanesulfonate (TEAOTf, >99%) was purchased from TCI America (Portland, OR, USA). Kryptofix^®^ 2.2.2 (K_222_, >99%), cesium carbonate (Cs_2_CO_3_, 99%), potassium carbonate (K_2_CO_3_, >99%), anhydrous pyridine (Py, 99.8%), anhydrous N,N-dimethylformamide (DMF, 99.8%), anhydrous N,N-dimethylacetamide (DMA, 99.8%), anhydrous dimethyl sulfoxide (DMSO, >99.9%), n-butanol (n-BuOH, 99.9%), 1,3-dimethyl-2-imidazolidinone (DMI, >99.5%), anhydrous acetonitrile (MeCN, 99.8%) and anhydrous ethyl alcohol (EtOH, >99.5%), polyethylene (PE) frit (product no. 57183) were purchased from Sigma-Aldrich (St. Louis, MO, USA). Deionized (DI) water was obtained from a Milli-Q water purification system (EMD Millipore Corporation, Berlin, Germany). C18 plus light cartridges (130 mg sorbent, WAT023501) were purchased from Waters Corporation (Milford, MA, USA). Reagent and collection vials were purchased from Eppendorf (Hamburg, Germany). Reaction vials (4 mL; part# CG-4904-06) for macroscale reactions were purchased from Chemglass Life Sciences (Vineland, NJ, USA). Silicone oil (CAS 63148-62-9) used in the vial heating block was purchased from Fisher Chemical (Pittsburgh, PA, USA). 50 mL polypropylene centrifuge tubes were purchased from Corning Inc. (430 304, Corning, NY, USA). TLC plates were purchased from Merck KGaA (Darmstadt, Germany). Plastic scintillator plate was purchased from Eljen Technology (Sweetwater, TX, USA). Oasis HLB plus short (cat. no. 186000132) was obtained from Waters Corporation (Milford, MA, USA). [^18^F]Fluoride in [^18^O]H_2_O was obtained from the UCLA Crump Cyclotron and Radiochemistry Center and the UCLA Biomedical Cyclotron Facility (Los Angeles, CA, USA). The activity was used directly as obtained from the cyclotron without further purification.

### 4.2. Preparation of the FVSB Precursor for Radiolabeling

The uronium precursor for [^18^F]FVSB was synthesized following the literature procedure (Scheme S1) [22]. Purification of the precursor was performed utilizing crystallization methods. Briefly, the crude precursor was dissolved in a minimum amount of CHCl_3_. To this solution, hexane was gradually added until saturation was achieved, as indicated by the formation of a white precipitate. The solution was placed in a freezer at −20 °C to facilitate crystal growth. A small amount of crystal was formed, primarily consisting of impurities, while the desired product remained in the mother liquid. The first crop of crystals was removed by filtration and discarded. The filtrate was evaporated partially, and a few drops of hexane were added to bring the solution close to its saturation point again. The solution was placed in a freezer at −20 °C, allowing pure crystalline product to form over time. The off-white crystals were filtered and dried under vacuum to remove residual solvent. The ^1^H, ^13^C, and ^19^F NMR spectroscopic data were consistent with previously reported values [22].

### 4.3. Preparation of ^19^F-Fluorinated Reference Standards

Synthesis of 1-fluoro-4-(vinylsulfonyl)benzene (FVSB reference standard) and the peptide conjugate reference standard was performed following literature protocols [22]. The ^1^H, ^13^C, and ^19^F NMR spectroscopic data for the synthesized compounds were consistent with previously reported values.

### 4.4. Droplet-Based Radiosynthesis and Optimization of [^18^F]FVSB

Droplet-based reactions were performed on silicon chips coated with Teflon, each featuring a 4 × 4 array of hydrophilic reaction sites (Figure 2A), fabricated as previously described [46] and mounted on a temperature-controlled heating platform (Figure 2B). The general synthetic procedure (Figure 2C) involved the following steps: A 10 μL aliquot of an [^18^F]fluoride stock solution (containing a specified amount of phase transfer catalyst (PTC) and base) was dispensed onto a reaction site using a micropipette and dried via sequential heating at 95 °C for 10 s, 100 °C for 20 s, and 105 °C for 30 s. Next, 10 μL of a precursor stock solution (containing the desired amount of precursor in the reaction solvent) was added using a micropipette and heated to the specified temperature for the desired time to perform the fluorination reaction. After reaction completion, the crude product was collected by adding 15 μL of a collection solution and transferring the mixture via micropipette into a 0.5 mL Eppendorf tube for further analysis. To minimize residual activity on the chip, the collection process was repeated a total of four times.

Optimization studies were conducted to evaluate the effects of base type and quantity, solvent type, reaction temperature, reaction duration, precursor quantity, and composition of collection solution. Fresh stock solutions were prepared before each set of experiments. PTC/base stock solutions were formulated in deionized (DI) water. The optimal quantities were determined during the study. The [^18^F]fluoride stock solution was prepared by mixing [^18^F]fluoride/[^18^O]H_2_O with the PTC/base stock solution, ensuring that each 10 μL portion contained ∼23 MBq [~620 µCi] of activity along with the required base amount for a single reaction. Just before synthesis, the precursor was dissolved in the desired reaction solvent(s) at a concentration that ensured that each 10 μL portion contained the desired amount of precursor. The collection solution was prepared by mixing MeCN and DI water in a 1:1 (*v*/*v*) ratio. After synthesis, [^18^F]FVSB was purified using a custom-made cartridge constructed from Teflon tubing packed with divinylbenzene–N-vinylpyrrolidone copolymer resin (Supplementary Materials, Section S4.4).

### 4.5. Radiochemical Analysis of [^18^F]FVSB

Radioactivity measurements were conducted using a calibrated dose calibrator (CRC-25R, Capintec, Florham Park, NJ, USA). The presence of both [^18^F]FVSB and radiotracer was confirmed by radio-HPLC using an analytical column (Luna C18, 250 × 4.6 mm, 5 μm; Agilent Technologies, Santa Clara, CA, USA) at a flow rate of 1 mL/min. Chromatographic separation was achieved with an initial mobile phase of 5:95 *v*/*v* MeCN/water (containing 0.1% TFA, *v*/*v*) for 3 min, followed by a linear gradient to 80:20 *v*/*v* MeCN/water(containing 0.1% TFA, *v*/*v*) over 27 min, and then a fixed ratio of 5:95 *v*/*v* MeCN/water (containing 0.1% TFA, *v*/*v*) over the next 10 min (for cleaning). The radio-HPLC system (Knauer Smartline, Berlin, Germany) was equipped with a degasser (Model 5050), pump (Model 1000), UV detector set at 254 nm (Eckert & Ziegler, Berlin, Germany), and a gamma detector (BFC-4100; Bioscan, Inc., Poway, CA, USA) coupled to a radiation counter (BFC-1000; Bioscan, Inc., Poway, CA, USA). Under these conditions, [^18^F]FVSB eluted at 23 min, whereas the ^18^F-labeled peptide showed a retention time of 16 min. Both products [^18^F]FVSB and ^18^F-labeled peptide were confirmed by co-injection with the reference standard and matched previous literature reports [22]. The same analytical radio-HPLC system was applied, except that the wavelength was changed to 240 nm to determine the molar activity of the purified prosthetic group and labeled peptide (Supplementary Materials, Section S5).

To enable high-throughput evaluation of reaction performance for optimization purposes, multiple samples were analyzed in parallel via multi-lane radio-thin layer chromatography (radio-TLC) techniques [47]. In brief, 0.5 μL of each sample was applied to TLC plates (6 cm × 5 cm sections cut from 20 cm × 5 cm silica gel 60 F254 sheets). The plates were developed to a length of 4 cm using a mobile phase of dichloromethane (DCM) and methanol (MeOH) in a 9:2 volume ratio. After drying, the plates were covered with a plastic scintillator plate (75 × 50 × 1 mm^3^) and subjected to scintillation imaging with a 5 min exposure time. Images were corrected, and then the radiochemical conversion (RCC) for each sample lane was determined using region of interest (ROI) analysis, as previously described [47]. The [^18^F]FVSB crude product consists of two bands, one at the origin (R_f_ = 0.07), which corresponds to [^18^F]fluoride (verified via spotting of [^18^F]fluoride in a separate lane), and a more nonpolar band (R_f_ = 0.91), which represents the product. Collection efficiency (Coll. Eff.) was calculated by dividing the recovered activity of the crude product mixture from a single reaction site by the initially loaded activity at that reaction site, with decay-correction applied. To calculate the radioactivity loaded to each reaction site, the total activity in the source vial was measured prior to loading and re-measured afterward. The decrease in activity reflected the amount loaded onto the site. The primary metric of reaction performance, i.e., crude RCY, was derived by multiplying RCC by the collection efficiency.

Finally, to help identify losses in each reaction, the residual activity on the chip after each crude product was collected and quantified. The total activity of the chip after collecting all crude products was first measured using a dose calibrator, covered with a scintillator plate, followed by scintillator imaging of the chip surface with a 5 min exposure. The fraction of activity associated with each reaction site was then determined by ROI analysis as previously described. These fractions were multiplied by the total activity on the chip to yield the residual activity at each individual reaction site. These values were used to compute the residual activity as a fraction of the initially loaded activity for each reaction, using decay correction.

### 4.6. Bioconjugation of [^18^F]FVSB with Cystine-Containing Peptide

Peptide bioconjugation with the prosthetic group was carried out on the 4 × 4 Teflon-coated silicon chips (Figure 5) using both a one-pot approach and a two-step process. In the one-pot approach, [^18^F]FVSB was synthesized on chip using the optimized conditions. A stock peptide solution was prepared in a 1:1 (*v*/*v*) mixture of the selected buffer and MeOH at the desired concentration. Next, a 10 µL peptide solution was loaded onto the crude [^18^F]FVSB using a micropipette, and the reaction was allowed to proceed at the selected temperature. Upon completion, the crude reaction mixture was recovered by washing each site with four aliquots of collection solution (4 × 15 μL), and the combined volume was transferred to a 0.5 mL Eppendorf tube for analysis by TLC. In the two-pot process, [^18^F]FVSB was synthesized following the same protocol and then subjected to cartridge purification. Purified [^18^F]FVSB was dispensed onto the chip as a series of aliquots (each dispensing step followed by evaporation of solvent), distributed across two new reaction sites. Subsequently, the peptide solution was applied to each reaction site using a micropipette, and the bioconjugation was allowed to proceed at the appropriate temperature. After completion of the reaction, the crude product was analyzed via TLC as described above. Optimization studies were carried out to evaluate the influence of the reaction time and temperature on the bioconjugation performance.

### 4.7. Radiochemical Analysis of ^18^F-Labeled RGDC Peptide

The radioactivity of the ^18^F-labeled RGDC peptide was quantified using a calibrated dose calibrator. The RCC of [^18^F]FVSB to ^18^F-labeled peptide was determined by multi-lane radio-TLC, performed similarly to analysis of [^18^F]FVSB samples, except the mobile phase was deionized water and MeCN (1:1, *v*/*v*) containing 0.1% TFA (*v*/*v*). The crude ^18^F-labeled peptide showed three bands on the TLC plate: the primary band corresponding to the desired radiotracer (Rf = 0.57), a second band representing an unidentified species—potentially an isomer of the radiotracer (Rf = 0.67), and a third band attributable to unreacted [^18^F]FVSB (Rf = 0.90). The possibility of an isomer is further supported by the HPLC chromatogram (Figure S17), which shows a shoulder peak whose characteristics (e.g., polarity) appear comparable to the FVSB-adjacent band (Rf = 0.67) observed on the TLC plate.

## 5. Conclusions

This study demonstrates a rapid synthetic and optimization methodology that produces the thiol-reactive prosthetic group [^18^F]FVSB using 4–10-fold less precursor, 8–200-fold less solvent, and 20–30× shorter reaction time compared to conventional methods. The RCY for the microscale process (59 ± 3%) is significantly higher than that in conventional vial-based synthesis (46 ± 4%). In addition, this droplet-based microscale bioconjugation method requires 3-fold less peptide, 25-fold less solvent, and 3× shorter time for the peptide bioconjugation reaction to RGDC. A significant advantage of droplet reactions is the ability to leverage a high-throughput technique where multiple reactions can be conducted simultaneously, enabling rapid and thorough optimization of multiple reaction parameters. This study also introduced the first two-pot synthesis in a droplet microreactor by using a micro-cartridge to perform the purification of the crude [^18^F]FVSB and then reloading the purified [^18^F]FVSB to new reaction sites on the microscale labeling chip. The results could be extended in a straightforward manner to label other thiol-functionalized peptides and biomolecules.

## Figures and Tables

**Table 1:** Comparison of [^18^F]FVSB synthesis conditions and performance, as well as conditions and performance of bioconjugation reactions of [^18^F]FVSB with substrates to form stable thiol-linked conjugates. N.R. = Not reported.

	This Work	Ma et al. [22]	Hu et al. [23]	Wang et al. [24]	Craig et al. [43]
Reaction format	Droplet	Vial	Vial	Vial	Vial
Synthesis of [^18^F]FVSB (prosthetic group)					
Number of repeats	4	10	1	1	1
Starting activity (MBq)	26–37	600–1300	560	560	50–10,000
Precursor amount (mg)	0.5	5	2	2	3.50
Precursor amount (μmol)	0.82	8.23	3.29	3.29	10
Precursor conc. (M)	0.082	0.008	0.041	0.027	0.008
Reaction volume (μL)	10	1000	80	120	1200
Reaction solvent	DMA	Butanone/EtOH (10:1 *v*/*v*)	MeCN	MeCN	DMI/*^n^*BuOH (2:1 *v*/*v*)
Reaction temp. (°C)	90	130	90	90	120
Reaction time (min)	1	30	20	20	30
RCC (%)	74	94	N.R.	N.R.	83
Method to analyze RCC	TLC	HPLC	HPLC	HPLC	UHPLC
Collection efficiency (%)	86	N.R.	N.R.	N.R.	N.R.
Crude RCY (%)	64	N.R.	41	N.R.	N.R.
RCY (%)	59	46	N.R.	N.R.	N.R. ^1^
Activity Yield (%)	51	32	N.R.	N.R.	18
Molar activity at EOS (GBq/µmol)	>45	≥106	N.R.	N.R.	N.R.
Bioconjugation reaction (between prosthetic group and biomolecule)					
Number of repeats	4	3	1	3	3
Starting activity (MBq)	220–250	14–20	110	74	50–500
Substrate	RGDC	RGDC	Amino acid	PSMA	TATE, RADfC
Substrate amount (mg)	0.50	3	2	0.2	0.5–3.0
Substrate amount (μmol)	1.11	6.66	16.51	0.51	N.R.
Substrate conc. (M)	0.11	0.01	0.082	0.003	N.R.
Reaction volume (μL)	10	500	200	200	1000–2000
Reaction temp. (°C)	55	35	35	85	35
Solvent/Buffer (All are 1:1 *v*/*v* mixtures)	HEPES (pH 7.3)/MeOH	Borate (pH 8.5)/MeOH	Borate (pH 8.5)/MeOH	Borate (pH 8.5)/MeOH	Borate (pH 8.5)/MeOH
Reaction time (min)	10	30	30	15	30
RCC (%)	74	89	97–99	N.R.	98–99
Method to analyze RCC	TLC	HPLC	HPLC	HPLC	UHPLC
Collection efficiency (%)	75	N.R.	N.R.	N.R.	N.R.
Crude RCY (%)	55	84	42–60	18 ± 1–53 ± 3 ^2^	N.R.
Overall synthesis of [^18^F]FVSB-conjugate					
Synthesis time up until purification (min)	46	N.R.	N.R.	N.R.	N.R.
Crude RCY (%)	37	N.R.	N.R.	N.R.	N.R.
Apparent molar activity at EOS (GBq/µmol)	0.588	N.R.	N.R.	N.R.	N.R.

^1^ RCY could not be computed from activity yield because the synthesis time was not reported. ^2^ A range of bioconjugation yields is listed using different prosthetic groups, and it is not clear which corresponds to [^18^F]FVSB.

## Data Availability

Data are contained within the article and Supplementary Materials.

## Supplementary Materials

The following supporting information can be downloaded at: https://www.mdpi.com/article/10.3390/molecules31111777/s1, Scheme S1. Synthesis of FVSB Precursor. Figure S1. Analytical HPLC chromatogram for purified (via cartridge) [^18^F]FVSB. Figure S2. Example analytical HPLC chromatogram for a co-injection of purified (via cartridge) [^18^F]FVSB with the FVSB reference standard. Table S1. Results of optimization of precursor amount for the microscale synthesis of [^18^F]FVSB. Table S2. Results of optimization of base amount for the microscale synthesis of [^18^F]FVSB. Figure S3. Experimental results from the study on the influence of base amount on the microscale synthesis of [^18^F]FVSB. Table S3. Results of optimization of base types for the microscale synthesis of [^18^F]FVSB. Figure S4. Experimental results from the study on the influence of base type on the microscale synthesis of [^18^F]FVSB. Table S4. Results of the optimization of reaction solvent for the microscale synthesis of [^18^F]FVSB. Figure S5. Experimental results from the study on the influence of reaction solvent on the microscale synthesis of [^18^F]FVSB. Figure S6. Impact of reaction solvent on the performance of the microscale synthesis of [^18^F]FVSB. Table S5. Results of the optimization of temperature for the microscale synthesis of [^18^F]FVSB. Figure S7. Experimental results from the study on the influence of temperature on the microscale synthesis of [^18^F]FVSB. Table S6. Results of the optimization of reaction time for the microscale synthesis of [^18^F]FVSB. Figure S8. Experimental results from the study on the influence of reaction time on the microscale synthesis of [^18^F]FVSB. Figure S9. Impact of reaction time on the performance of the microscale synthesis of [^18^F]FVSB. Table S7. Results of the optimization of the collection solvent for the microscale synthesis of [^18^F]FVSB. Figure S10. Experimental results from the study on the influence of collection solvent on the microscale synthesis of [^18^F]FVSB. Figure S11. Impact of collection solvent on the performance of the microscale synthesis of [^18^F]FVSB. Solvent mixtures are all *v*/*v*. Table S8. Results of optimization of reaction time on the microscale bioconjugation reaction between [^18^F]FVSB and RGDC peptide. Figure S12. Experimental results from the study on the influence of bioconjugation time on the microscale synthesis of ^18^F-labeled RGDC peptide. Figure S13. Impact of bioconjugation time on the microscale synthesis of ^18^F-labeled RGDC peptide. Table S9. Results of the optimization of reaction temperature for the microscale bioconjugation reaction between [^18^F]FVSB and RGDC peptide. Figure S14. Experimental results from the study on the influence of bioconjugation temperature on the microscale synthesis of ^18^F-labeled RGDC peptide. Figure S15. Impact of bioconjugation temperature on performance of the microscale synthesis of ^18^F-labeled RGDC peptide. Table S10. Results of one-pot synthesis of [^18^F]FVSB and conjugation to RGDC. Table S11. Results of purification of [^18^F]FVSB through a custom-made micro cartridge. Figure S16. Preparation of a microcartridge for the purification of [^18^F]FVSB. Figure S17. Example of analytical HPLC chromatogram for crude ^18^F-labeled RGDC peptide produced using the optimized microscale synthesis. Figure S18. TLC analysis of crude [^18^F]FVSB and crude ^18^F-labeled RGDC peptide. Figure S19. Example analytical HPLC chromatogram for a co-injection of HPLC-purified ^18^F-labeled RGDC peptide with the reference standard. Figure S20. Calibration curve for molar activity determination of [^18^F]FVSB. Figure S21. Example analytical HPLC chromatogram of HPLC-purified [^18^F]FVSB for molar activity determination. Figure S22. Calibration curve for molar activity determination of ^18^F-labeled RGDC peptide. Figure S23. Example analytical HPLC chromatogram of HPLC-purified ^18^F-labeled RGDC peptide for determination of molar activity. Figure S24. ^1^H NMR spectrum of FVSB precursor (7) in CDCl_3_. Figure S25. ^13^C NMR spectrum of FVSB precursor (7) in DMSO; Figure S26. ^1^H NMR spectrum of FVSB reference standard in CDCl_3_; Figure S27. ^19^F NMR spectrum of FVSB reference standard in CDCl_3_. Figure S28. ^13^C NMR spectrum of FVSB reference standard in CDCl_3_.

## Abbreviations

The following abbreviations are used in this manuscript:

DCM	Dichloromethane
DI water	Deionized water
DMA	Dimethylacetamide
DMI	1,3-dimethyl-2-imidazolidinone
DMSO	Dimethyl sulfoxide
FVSB	Fluoro-4-(vinylsulfonyl)benzene
HPLC	High-performance liquid chromatography
K_222_	Kryptofix 2.2.2
PTC	Phase transfer catalyst
RCC	Radiochemical conversion
RCY	Radiochemical yield
RGDC	Arg-Gly-Asp-Cys (peptide)
ROI	Region of interest
TFA	Trifluoro acetic acid
TLC	Thin-layer chromatography
UHPLC	Ultra high-performance liquid chromatography
