# Supplementary material for: Droplet-Based Radiosynthesis and High-Throughput Optimization of Vinyl Sulfone Prosthetic Group ([18F]FVSB) and Peptide Bioconjugation
Source: Molecules. 2026 May 22;31(11):1777. doi: 10.3390/molecules31111777 (PMC13257646; doi:10.3390/molecules31111777)
Supplement: Supplementary file 1 [file molecules-31-01777-s001.zip › molecules-4233146-supplementary.pdf]

Table of Contents

1. General chemistry materials and methods 3
   1.1 Reagents and materials 3
   1.2 Analytical equipment and methods 3
2. Experimental Procedure and Characterization Data 4
   2.1 Radiochemical analysis of [^{18}F]FVSB 5
3. Optimization of [^{18}F]FVSB synthesis 6
   3.1 Influence of precursor amount 6
   3.2 Effect of base amount 6
   3.3 Influence of PTC/base type 7
   3.4 Influence of reaction solvent 8
   3.5 Effect of temperature 10
   3.6 Influence of reaction time 11
   3.7 Influence of Collection Solvent 13
4. Optimization of ^{18}F-labeling of RGDC peptide 14
   4.1 Optimization of Bioconjugation Time 14
   4.2 Optimization of bioconjugation temperature 16
   4.3 Overall synthesis of ^{18}F-labeled RGDC peptide in one-pot process 18
   4.4 Purification of [^{18}F]FVSB 18
   4.5 Analysis of ^{18}F-labeled RGDC peptide 20
5. Molar Activity 22
6. NMR spectra of synthesized compounds 25
7. References 27

1. General chemistry materials and methods

1.1 Reagents and materials

All chemicals and reagents were obtained from commercial suppliers and used as they received without additional purification. Chloroimidazolium chloride was purchased from Strem Chemicals, Inc. and utilized without modification. Peptides were acquired from Bachem Americas, Inc. and used as supplied. A silicone oil bath served as the heating medium for all non-radioactive reactions. Deuterated solvents were purchased from Cambridge Isotope Laboratories. Unless specified otherwise, reactions were performed in oven-dried glassware with commercially available anhydrous solvents. Solvents employed for extraction and chromatography were not anhydrous. Reaction monitoring and chromatography fractions were analyzed using thin-layer chromatography (TLC) on Merck precoated silica gel 60 F_{254} glass plates (250 μm), with visualization achieved through ultraviolet irradiation. Flash column chromatography was carried out using E. Merck silica gel 60 (230−400 mesh) under compressed air.

1.2 Analytical equipment and methods

Nuclear magnetic resonance (NMR) spectra were recorded on a Bruker ARX 400 spectrometer (operating at 400 MHz for ^{1}H, 100 MHz for ^{13}C, and 376 MHz for ^{19}F). Chemical shifts are expressed in parts per million (ppm, δ), using residual solvent peaks as internal references. Coupling constants (J) are provided in Hertz (Hz), and signal multiplicities are designated as follows: br (broad), s (singlet), d (doublet), t (triplet), q (quartet), hept (heptet), and m (multiplet).

2. Experimental Procedure and Characterization Data

Scheme S 1.Synthesis of FVSB precursor[α]

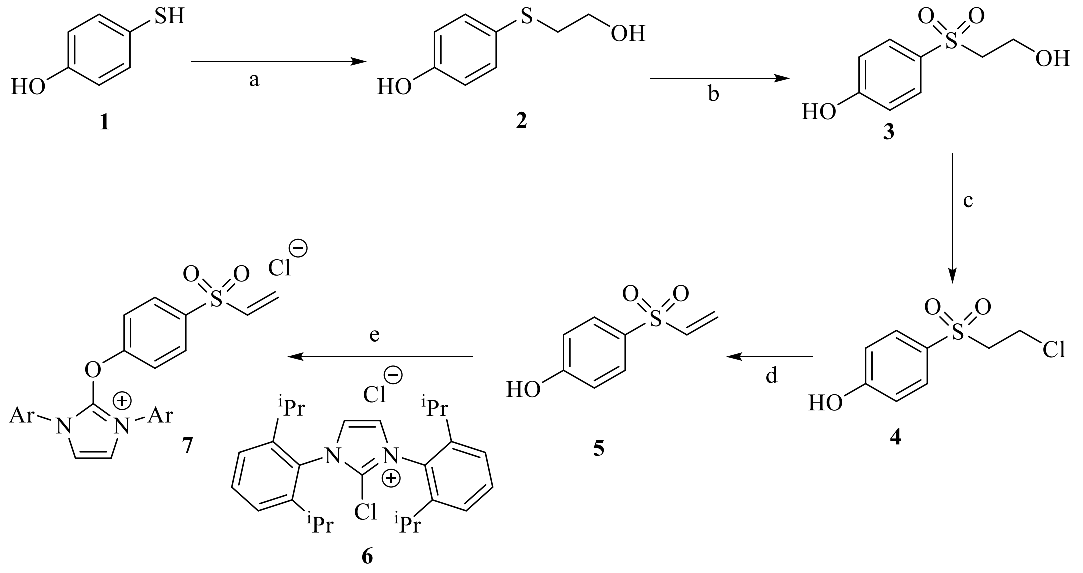

[α]Reagents and conditions: (a) 2-bromoethanol, 1.0 N aq. NaOH, MeOH, 23 °C, 21 h; (b) Oxone, MeOH, 23 °C, 2 h; (c) $SOCl_2$, pyridine, CH_2Cl_2, 23 °C, 20 h; (d) Et_3N, THF, 23 °C, 24 h; (e) Ag_2CO_3, $CHCl_3$, 60 °C, 4.5 h.

2.1 Radiochemical analysis of [^{18}F]FVSB

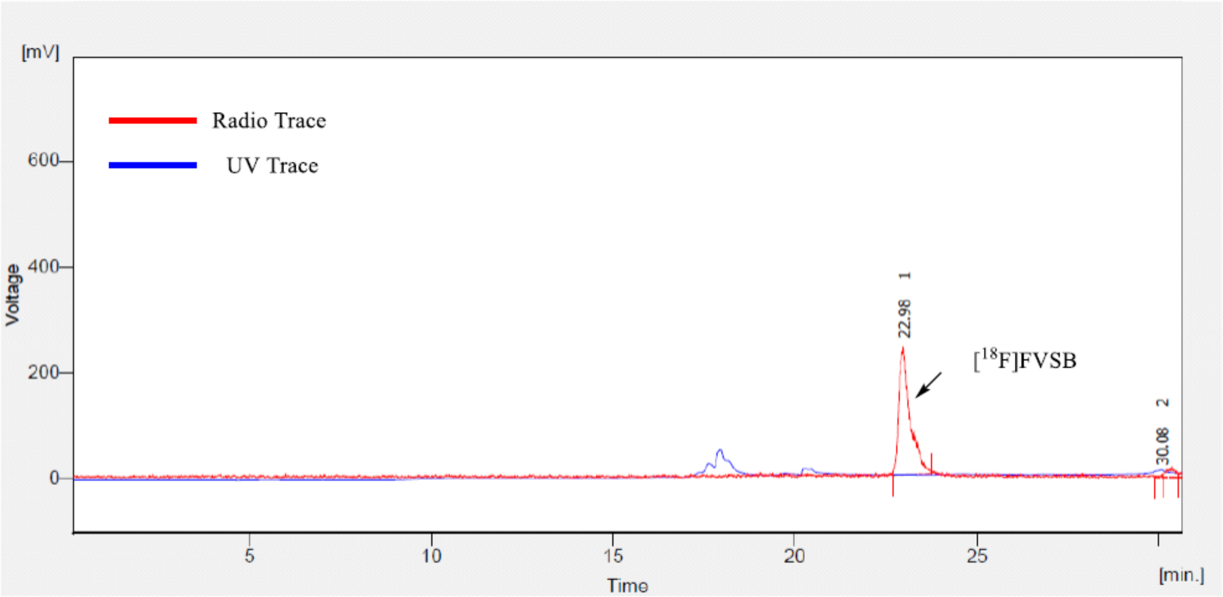

Figure S1. Example analytical HPLC chromatogram for purified (via cartridge) [^{18}F]FVSB.

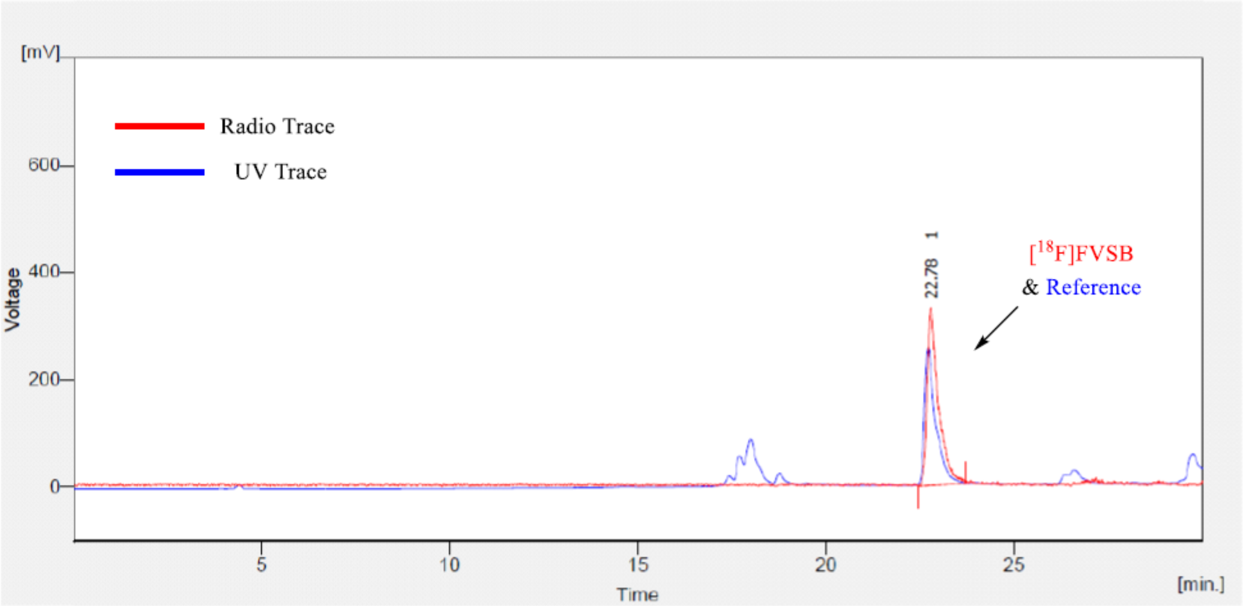

Figure S2. Example analytical HPLC chromatogram for a co-injection of purified (via cartridge) [^{18}F]FVSB with the FVSB reference standard.

3. Optimization of [^{18}F]FVSB synthesis

3.1 Influence of precursor amount

Table S1. Results of optimization of precursor amount for the microscale synthesis of [^{18}F]FVSB[a]

Precursor amount (mg)	Coll. Eff. (%)	RCC (%)	RCY (%)	Activity left on chip (%)
1.00	90 ± 6	41 ± 2	37 ± 2	0.4 ± 0.5
0.75[b]	94 ± 4	42 ± 6	38 ± 3	0.1 ± 0.0
0.50	91 ± 5	44 ± 2	39 ± 4	0.1 ± 0.0
0.25	84 ± 4	41 ± 1	34 ± 2	0.1 ± 0.0
0.05	91 ± 3	8 ± 4	7 ± 3	0.1 ± 0.0

[a] All reactions were performed using $TEAHCO_3$ (0.05 mg, 0.261 µmol) during the F-18 drying step, and then adding the indicated amount of precursor in 10 µL of DMA and reacting at 90 °C for 1 min to achieve fluorination (n = 4 replicates). [b]n = 2 replicates.

3.2 Effect of base amount

Table S2. Results of optimization of base amount for the microscale synthesis of [^{18}F]FVSB[a]

Base mass (mg)	Coll eff (%)	RCC (%)	RCY (%)	Activity left on chip (%)
1.00 [b]	89 ± 5	6 ± 1	5 ± 1	0.4 ± 0.2
0.75 [b]	93 ± 2	6 ± 1	5 ± 1	0.5 ± 0.2
0.50	87 ± 8	14 ± 3	12 ± 4	0.6 ± 0.1
0.10 [b]	89 ± 2	52 ± 2	46 ± 2	0.7 ± 0.1
0.05	86 ± 3	47 ± 3	40 ± 3	0.8 ± 0.1

[a]All reactions were performed using indicated amount of $TEAHCO_3$ during the F-18 drying step, and then adding 0.5 mg (0.82 µmol) of precursor in 10 µL of DMA and reacting at 90 °C for 1 min to achieve fluorination (n = 4 replicates unless otherwise specified). [b]n = 3 replicates.

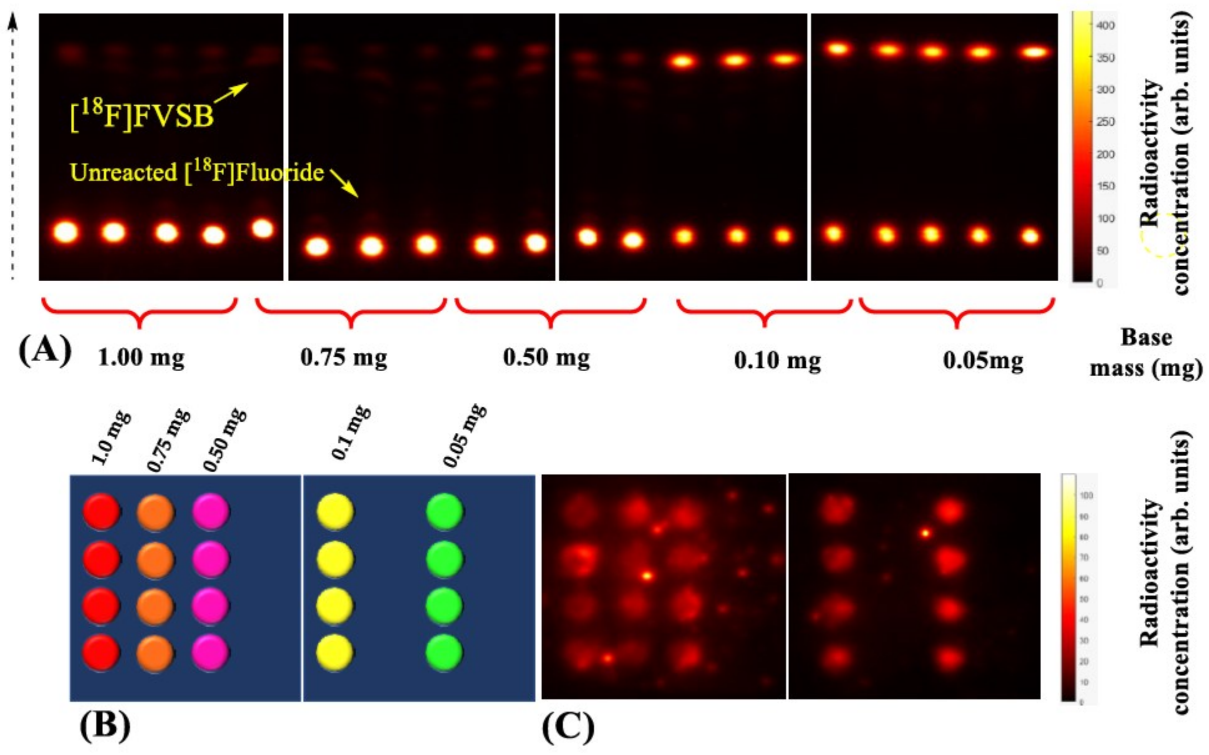

Figure S3. Experimental results from study of influence of base amount on the microscale synthesis of [^{18}F]FVSB. **A.** High-throughput analysis of crude [^{18}F]FVSB using multi-lane TLC with scintillation imaging readout. **B.** Map of conditions at reaction sites. **C.** Scintillation images of chips after collection of crude products showing the distribution of residual activity.

3.3 Influence of PTC/base type

Table S 3. Results of optimization of base types for the microscale synthesis of [^{18}F]FVSB [a]

Types of Bases	Coll. Eff. (%)	RCC (%)	RCY (%)	Activity left on chip (%)
$TEAHCO_3$	78 ± 13	63 ± 9	49 ± 13	0.5 ± 0.2
$Cs_2CO_3 + K_{222}$ [b]	77 ± 12	6 ± 6	5 ± 4	0.7 ± 0.4
$K_2CO_3 + K_{222}$ [b]	75 ± 6	10 ± 5	7 ± 4	3.2 ± 1.0
TEAOTf	82 ± 6	5 ± 3	4 ± 3	2.2 ± 0.5
TBAOTf	19 ± 2	1 ± 0	0 ± 0	2.6 ± 0.3

[a] All reactions were performed using the investigated base (0.1 mg) during the F-18 drying step, and then adding 0.5 mg (0.82 µmol) of precursor in 10 µL of DMA and reacting at 90 °C for 1 min to achieve fluorination (n = 4 replicates unless otherwise indicated). [b] n = 3 replicates.

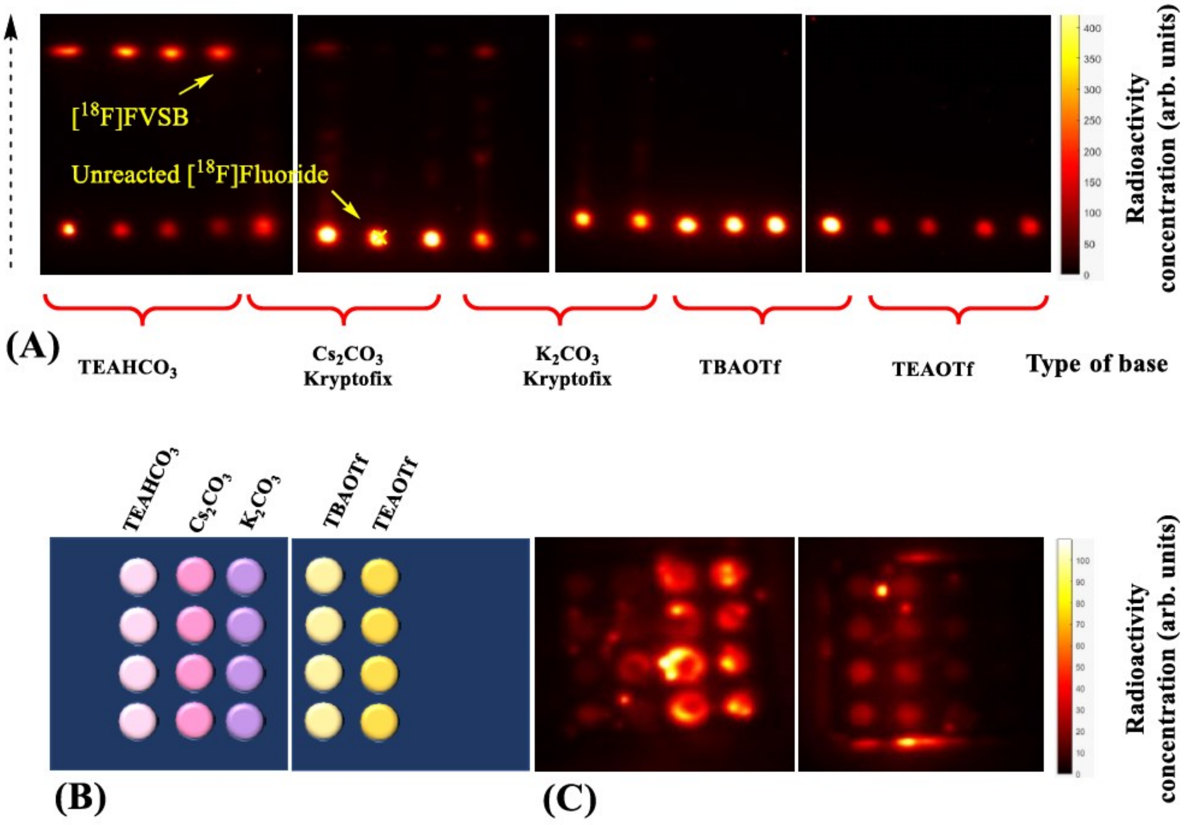

Figure S4. Experimental results from study of influence of base type on the microscale synthesis of [^{18}F]FVSB. **A.** High-throughput analysis of crude [^{18}F]FVSB using multi-lane TLC with scintillation image readout. **B.** Map of conditions at reaction sites **C.** Scintillation images of chips after collection of crude products showing the distribution of residual activity

3.4 Influence of reaction solvent

Table S4. Results of the optimization of reaction solvent for the microscale synthesis of [^{18}F]FVSB[a]

Solvent	Coll. Eff. (%)	RCC (%)	RCY (%)	Activity left on Chip (%)
DMI + pyridine (24:1 v/v)	95 ± 3	25 ± 3	24 ± 2	0.3 ± 0.1
DMF	90 ± 6	57 ± 5	51 ± 1	0.3 ± 0.2
MeCN + DMSO (3:1 v/v)	61 ± 3	33 ± 1	20 ± 2	0.7 ± 0.3
DMA	86 ± 5	59 ± 5	52 ± 5	0.4 ± 0.4
2 -butanone +EtOH (10:1 v/v)[b]	79 ± 0	57 ± 5	44 ± 5	

[a]All reactions were performed using $TEAHCO_3$ (0.1 mg, 0.52 µmol) during the F-18 drying step, and then adding 0.5 mg (0.82 µmol) of precursor in 10 µL of investigating solvent and reacting at 90 °C for 1 min to achieve fluorination (n = 4 replicates). [b]Residual activity data on the chip is

unavailable for this experiment, as the chip was damaged during detachment from the heating platform.

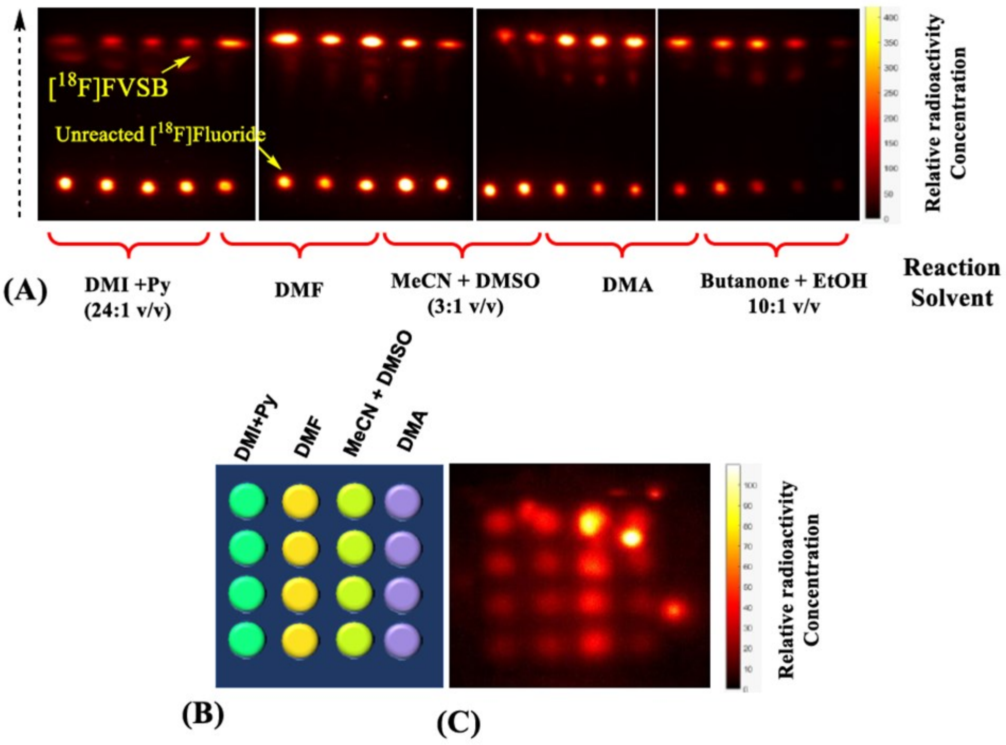

Figure S5. Experimental results from study of influence of reaction solvent on the microscale synthesis of [^{18}F]FVSB **A.** High-throughput analysis of crude [^{18}F]FVSB using multi-lane TLC with scintillation image readout. **B.** Map of conditions at reaction sites **C.** Scintillation images of chips after collection of crude products showing the distribution of residual activity.

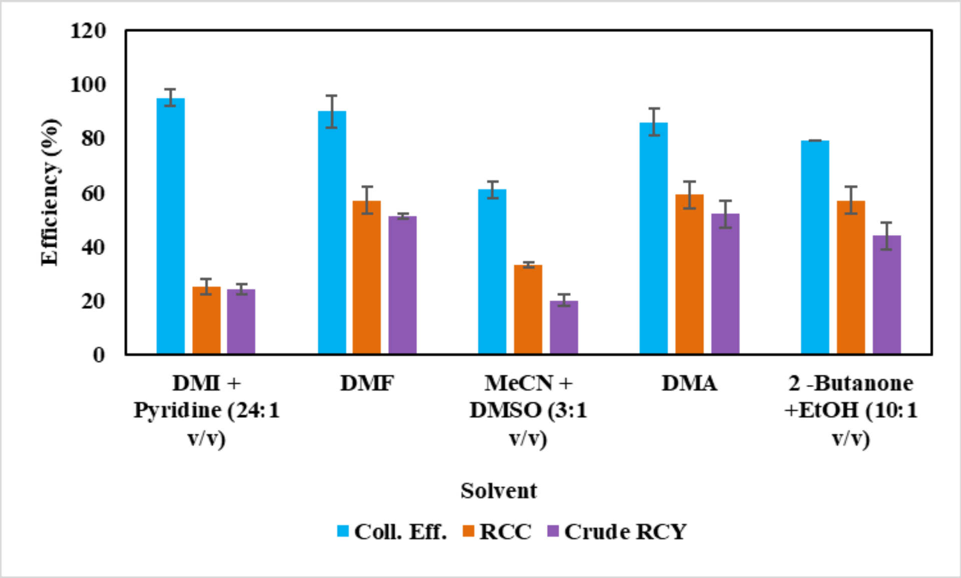

Figure S6. Impact of reaction solvent on the performance of the microscale synthesis of [^{18}F]FVSB.

3.5 Effect of temperature

Table S5. Results of the optimization of temperature for the microscale synthesis of [^{18}F]FVSB[a]

Temperature (°C)	Coll. Eff. (%)	RCC (%)	RCY (%)	Activity left on Chip (%)
60	86 ± 8	21 ± 2	18 ± 3	0.1 ± 0.0
70[b]	85 ± 2	34 ± 3	31 ± 6	0.2 ± 0.0
80	86 ± 4	54 ± 6	46 ± 7	0.2 ± 0.0
90	90 ± 7	63 ± 5	57 ± 8	0.2 ± 0.1
100	83 ± 6	66 ± 4	55 ± 4	0.2 ± 0.0
110	81 ± 7	68 ± 6	55 ± 9	0.2 ± 0.1
120	79 ± 4	70 ± 5	55 ± 2	0.4 ± 0.3

[a] All reactions were performed using $TEAHCO_3$ (0.1 mg, 0.52 µmol) during the F-18 drying step, and then adding 0.5 mg (0.82 µmol) of precursor in 10 µL of DMA and reacting for 1 min to achieve fluorination (n = 4 replicates unless otherwise specified). [b] n = 3 replicates.

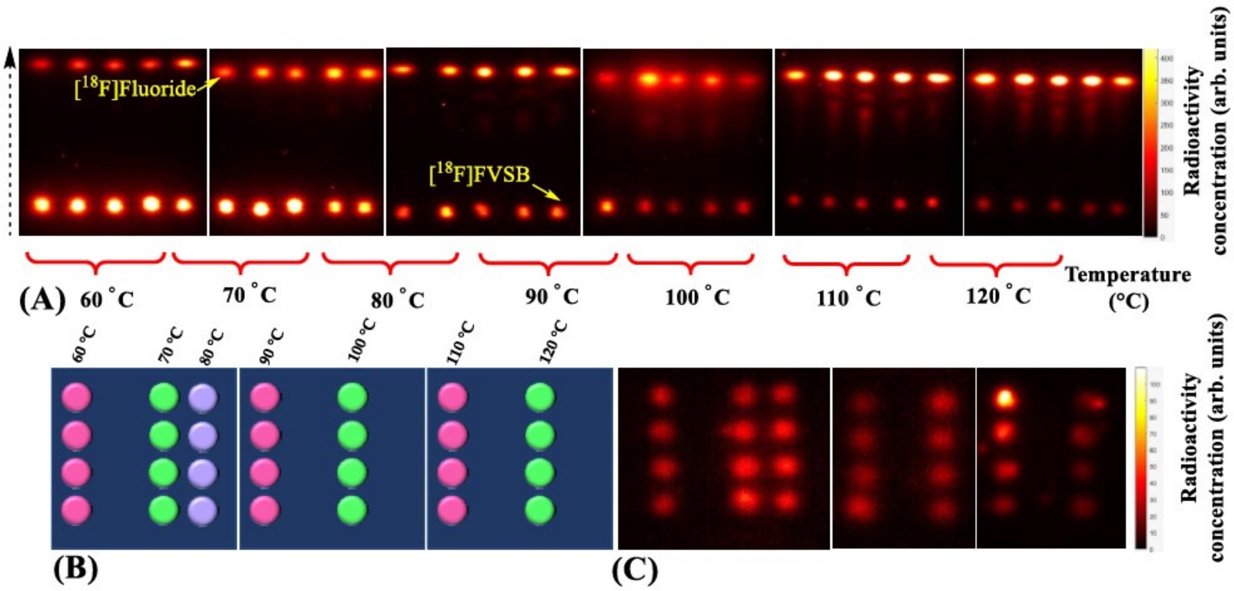

Figure S7. Experimental results from study of influence of temperature on the microscale synthesis of [^{18}F]FVSB. **A** High-throughput analysis of crude [^{18}F]FVSB using multi-lane TLC with scintillation image readout. **B.** Map of conditions at reaction sites **C.** Scintillation images of chips after collection of crude products showing the distribution of residual activity.

3.6 Influence of reaction time

Table S6. Results of the optimization of reaction time for the microscale synthesis of [^{18}F]FVSB[a]

Reaction time (min)	Coll. Eff. (%)	RCC (%)	RCY (%)	Activity left on chip (%)
0.5	88 ± 8	66 ± 8	58 ± 10	0.6 ± 0.4
1	85 ± 6	63 ± 6	54 ± 7	0.5 ± 0.1
2	76 ± 5	73 ± 7	56 ± 8	0.1 ± 0.0
3	85 ± 4	71 ± 1	60 ± 2	0.3 ± 0.1
4	76 ± 5	74 ± 2	56 ± 3	0.6 ± 0.3
5	77 ± 6	72 ± 4	55 ± 9	0.3 ± 0.1

[a]All reactions were performed with 0.1 mg (0.52 µmol) of $TEAHCO_3$ and then adding 0.5 mg (0.82 µmol) of precursor in 10 µL of DMA and reacting at 90 °C to achieve fluorination (n = 4 replicates).

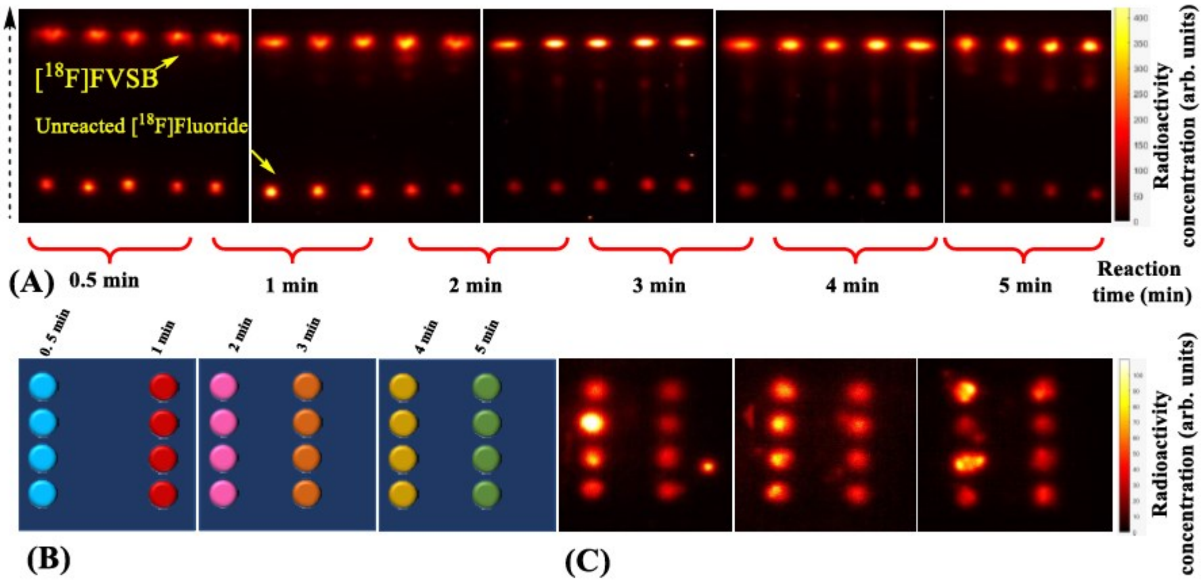

Figure S8. Experimental results from study of influence of reaction time on the microscale synthesis of [^{18}F]FVSB **A.** High-throughput analysis of crude [^{18}F]FVSB using multi-lane TLC with scintillation image readout. **B.** Map of conditions at reaction sites **C.** Scintillation images of chips after collection of crude products showing the distribution of residual activity.

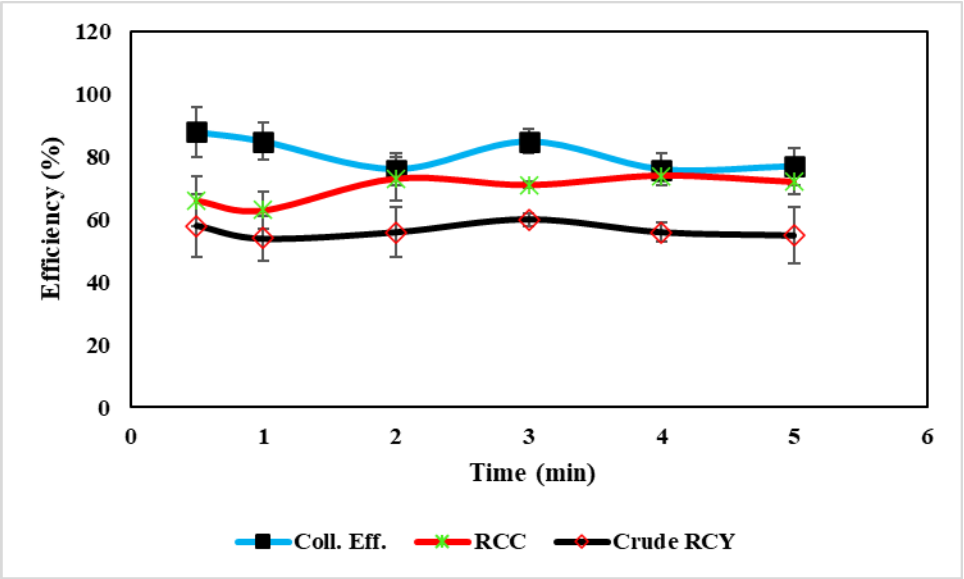

Figure S9. Impact of reaction time on the performance of the microscale synthesis of [^{18}F]FVSB.

3.7 Influence of Collection Solvent

Table S7. Results of the optimization of collection solvent for the microscale synthesis of [^{18}F]FVSB[a]

Collection Solvent	Coll. Eff. (%)	RCC (%)	RCY (%)	Activity left on chip (%)
MeCN:H_2O (1:1 v/v)	76 ± 12	60 ± 3	46 ± 9	0.1 ± 0.0
MeOH:H_2O (9:1 v/v)	71 ± 4	65 ± 2	46 ± 2	0.1 ± 0.0
MeOH:DCM (9:2 v/v)[b]	75 ± 4	63 ± 6	48 ± 4	0.7 ± 0.3
EtOH:H_2O (9:1 v/v)[b]	70 ± 10	60 ± 5	41 ± 9	0.4 ± 0.1
EtOH:DCM (9:2 v/v)	72 ± 2	41 ± 12	30 ± 9	0.1 ± 0.1
MeCN:MeOH (1:4 v/v) [b]	80 ± 5	48 ± 7	36 ± 4	0.5 ± 0.2

[a] All reactions were performed using $TEAHCO_3$ (0.1 mg, 0.52 µmol) during the F-18 drying step and then adding 0.5 mg (0.82 µmol) of precursor in 10 µL of DMA and reacting at 90 °C for 1 min to achieve fluorination (n = 4 replicates unless otherwise specified). [b] n = 3 replicates.

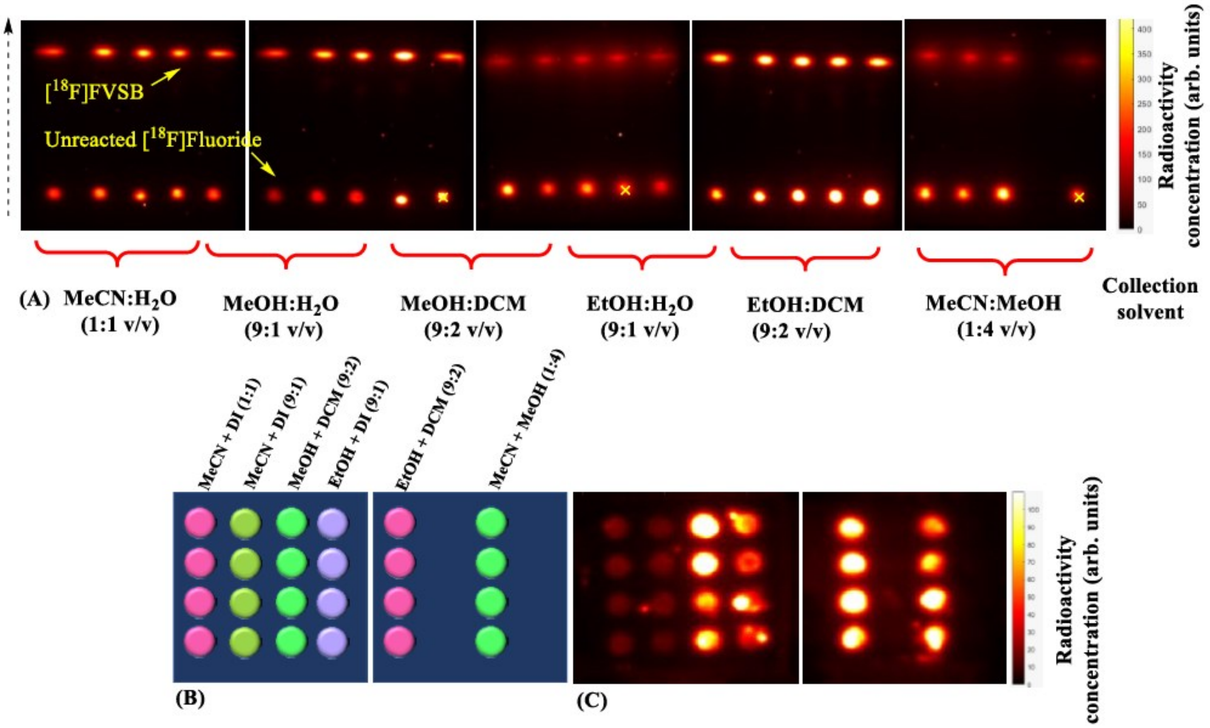

Figure S10. Experimental results from study of influence of collection solvent on the microscale synthesis of [^{18}F]FVSB **A**. High-throughput analysis of crude [^{18}F]FVSB using multi-lane TLC with scintillation image readout. **B.** Map of conditions at reaction sites **C.** Scintillation images of chips after collection of crude products showing the distribution of residual activity.

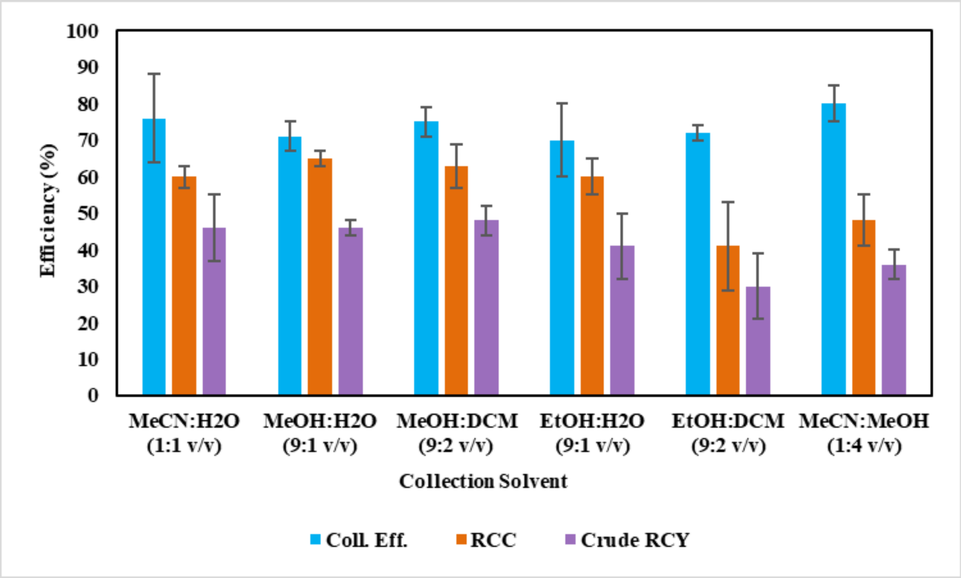

Figure S11. Impact of collection solvent on the performance of the microscale synthesis of [^{18}F]FVSB. Solvent mixtures are all v/v.

4. Optimization of ^{18}F-labeling of RGDC peptide

4.1 Optimization of Bioconjugation Time

Table S 8. Results of optimization of reaction time on the microscale bioconjugation reaction between [^{18}F]FVSB and RGDC peptide [a]

Bioconjugation Time (min)	Coll. Eff. (%)	RCC (%)	RCY (%)	Activity left on chip (%)
4	86 ± 4	13 ± 3	11 ± 3	0.37 ± 0.20
8 [b]	83 ± 8	15 ± 1	13 ± 2	0.32 ± 0.17
12	82 ± 9	18 ± 4	15 ± 5	0.70 ± 0.36
16	82 ± 6	21 ± 2	17 ± 2	0.34 ± 0.21
20 [b]	83 ± 2	21 ± 3	18 ± 3	0.61 ± 0.65

[a] All reactions were performed using $TEAHCO_3$ (0.1 mg, 0.52 µmol)) during the F-18 drying step, and then adding 0.5 mg (0.82 µmol) of precursor in 10 µL of DMA and reacting at 90 °C for 1 min to achieve fluorination (n = 4 replicates unless otherwise specified). Bioconjugation performed with 0.5 mg (1.11 µmol) of RGDC peptide at 37 °C. [b] n = 3 replicates.

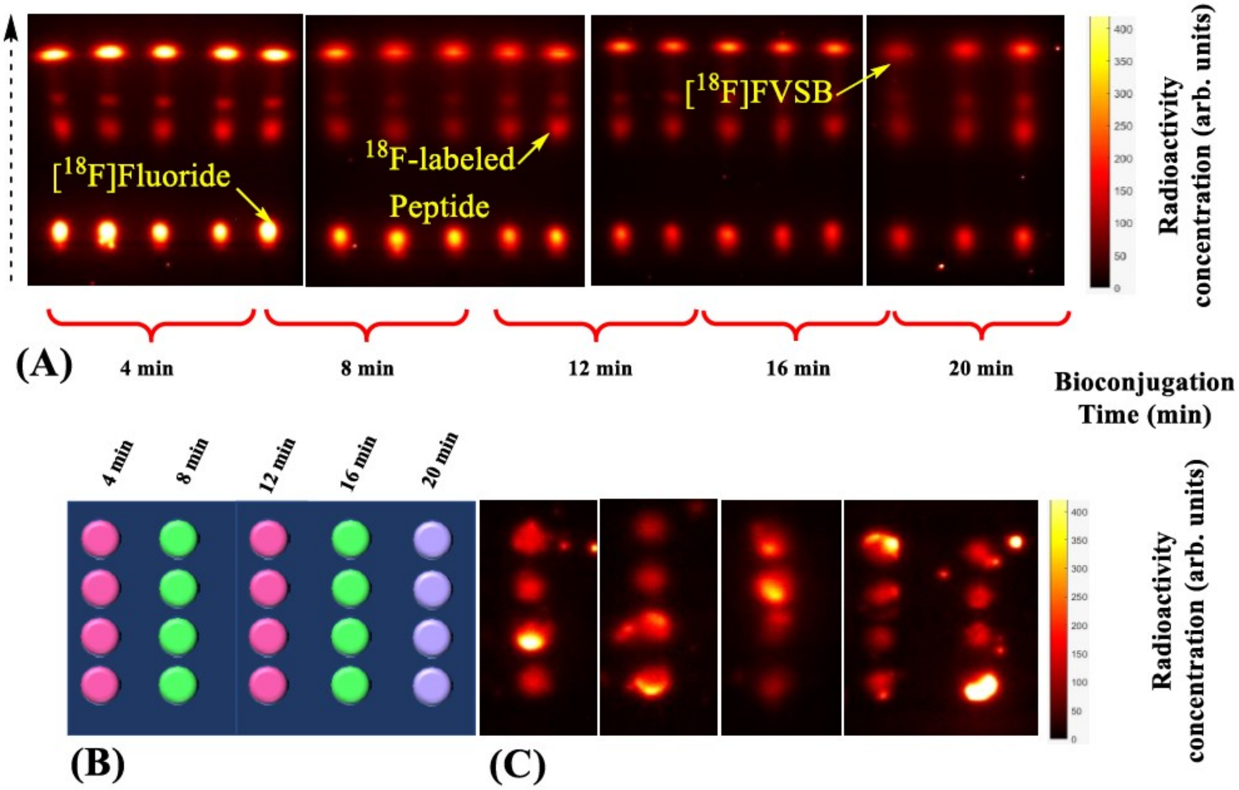

Figure S 12. Experimental results from study of influence of bioconjugation time on the microscale synthesis of the ^{18}F-labeled RGDC peptide **A.** High-throughput analysis of crude ^{18}F-labeled RGDC peptide using multi-lane TLC with scintillation image readout. **B.** Map of conditions at reaction sites **C.** Scintillation images of chips after collection of crude products showing the distribution of residual activity.

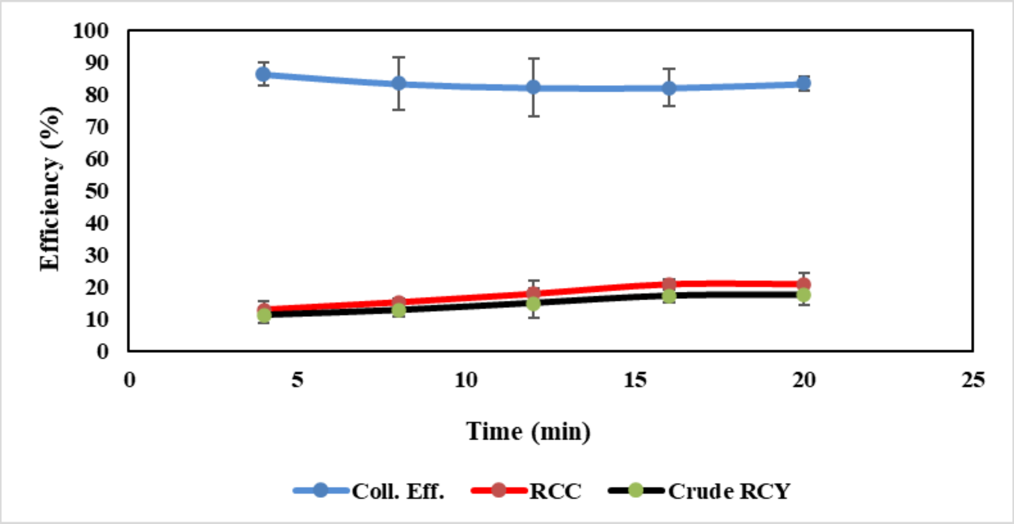

Figure S 13. Impact of bioconjugation time on the microscale synthesis of ^{18}F-labeled RGDC peptide.

4.2 Optimization of bioconjugation temperature

Table S 9. Results of the optimization of reaction temperature for the microscale bioconjugation reaction between [^{18}F]FVSB and RGDC peptide[a]

Temp. (°C)	Coll. Eff. (%)	RCC (%)	RCY (%)	Activity left on chip (%)
40	84 ± 4	21 ± 3	17 ± 1	1.58 ± 0.50
45	86 ± 4	22 ± 2	19 ± 1	0.76 ± 0.15
50	88 ± 3	22 ± 2	19 ± 2	0.71 ± 0.41
55 [b]	86 ± 2	24 ± 1	21 ± 1	0.91 ± 0.19

[a] All reactions were performed using $TEAHCO_3$ (0.1 mg, 0.52 µmol)) during the F-18 drying step, and then adding 0.5 mg (0.82 µmol) of precursor in 10 µL of DMA and reacting at 90 °C for 1 min to achieve fluorination (n = 4 replicates unless otherwise specified). Bioconjugation performed with 0.5 mg (1.11 µmol) of RGDC peptide in 10 µL of 1:1 (v/v) mixture of methanol and HEPES buffer (pH 7.3) for 10 min. [b] n = 3 replicates.

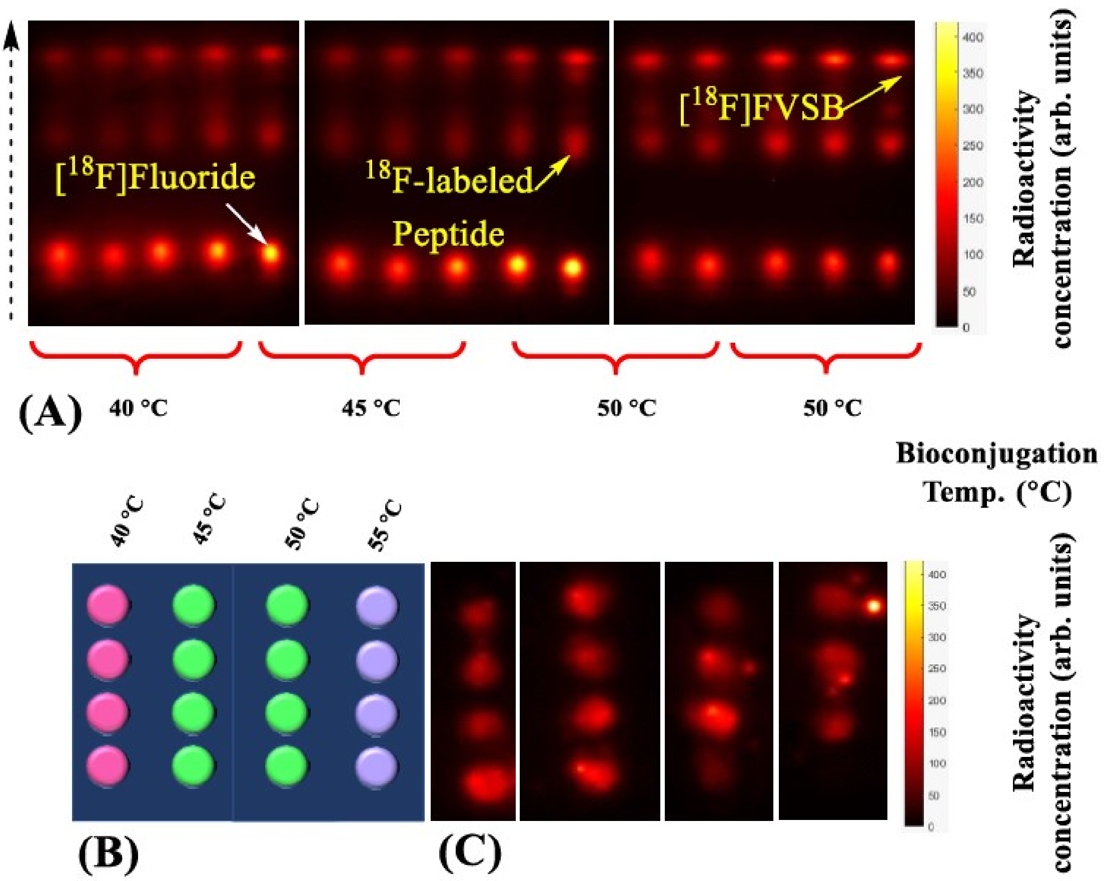

Figure S 14. Experimental results from study of influence of bioconjugation temperature on the microscale synthesis of ^{18}F-labeled RGDC peptide **A.** High-throughput analysis of crude ^{18}F-labeled RGDC peptide using multi-lane TLC with scintillation image readout. **B.** Map of conditions at reaction sites **C.** Scintillation images of chips after collection of crude products showing the distribution of residual activity

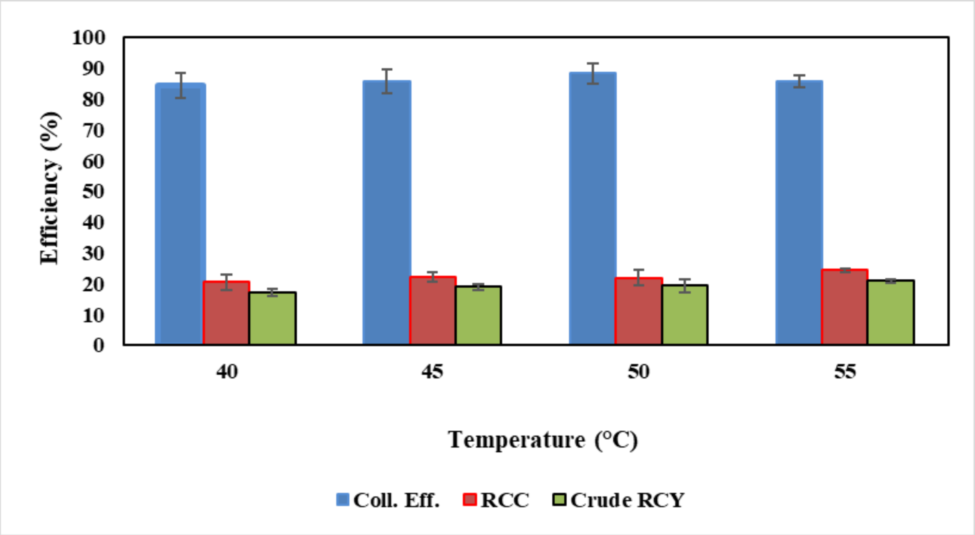

Figure S 15. Impact of bioconjugation temperature on performance of the microscale synthesis of ^{18}F-labeled RGDC peptide.

4.3 Overall synthesis of ^{18}F-labeled RGDC peptide in one-pot process

Table S 10. Results of optimized one-pot synthesis of [^{18}F]FVSB and conjugation to RGDC

Expt#	Coll. Eff. (%)	RCC (%)	Crude RCY (%)	Activity left on chip (%)
1	83	21	18	0.01
2	83	26	22	0.51
3	86	26	22	0.40
4	88	24	21	0.37
5	84	27	23	0.35
6	79	26	20	0.45
7	82	27	22	0.54
8	81	25	20	0.50
9	78	29	23	0.51
**Average**	**83 ± 3**	**26 ± 2**	**21 ± 2**	**0.40 ± 0.16**

4.4 Purification of [^{18}F]FVSB

During the synthesis of [^{18}F]FVSB under optimal conditions, impurities such as, a fraction of unreacted [^{18}F]fluoride remained. To purify the crude [^{18}F]FVSB prior to subsequent reactions, a cartridge-based purification strategy was employed. After purification, [^{18}F]FVSB was added back to the chip. Since the maximum volume that could be loaded on a reaction site was 20 µL, the full amount of purified [^{18}F]FVSB had to be loaded as a series of multiple aliquots each followed by solvent evaporation. Therefore, a major objective was to minimize the final volume of the purified

product to less than 200 µL in order to reduce the number of aliquots (and therefore the time needed) for activity transfer.

To meet this requirement, a custom cartridge (**Figure S18**) was fabricated from a 6–8 cm length of Teflon tubing (1/16″ inner diameter, 1/8″ outer diameter) as described previously [1]. A small piece of polyethylene frit of pore size 20 µm was inserted approximately 3 cm inside the tube, and the tubing was pinched at the frit position with pliers to create a narrow-diameter region. The sorbent bed, comprising divinylbenzene–N-vinylpyrrolidone copolymer resin (OASIS HLB), was then packed into the tube under vacuum as a slurry. A second frit was inserted to secure the packing, and the opposite end of the tube was pinched to firmly hold the sorbent bed in place.

Since resin mass and flow rate contribute to the performance of the trapping on the HLB resin, an optimization study of resin mass was conducted to assess trapping efficiency. Four cartridges were constructed, each preconditioned with 100 µL of ethanol followed by 500 µL of deionized water. Subsequently, 1 mL of deionized water was passed through the cartridge under nitrogen pressure (10 kPa) to mimic trapping of crude product. Cartridges containing 13 mg, 20 mg, 30 mg, and 40 mg of resin required 3, 4, 11, and 21 min, respectively, to pass 1 mL of water, clearly indicating that higher resin loading significantly slows the trapping process.

When the crude [^{18}F]FVSB was passed through a cartridge containing 13 mg of resin, the process took slightly longer (~10 min), potentially due to the presence of impurities. Measurement of cartridge and waste activities indicated a trapping efficiency of ~70% ± 3% (n = 4). After drying the cartridge with nitrogen to remove residual aqueous phase, sequential elution with 60 µL methanol, followed by an additional 60 µL, yielded an elution efficiency of ~98% ± 2% (n = 4). Based on these results, the cartridge with 13 mg of resin (4 cm resin length) was selected as the optimal design.

Table S 11. Results of purification of [^{18}F]FVSB through custom made micro cartridge.

Initial activity in source vial (A_0) (mCi)[a]	Activity in waste after trapping (A_{waste}) (mCi)[a]	Residual activity in source vial after trapping ($A_{0\_residual}$) (mCi)[a]	Eluted activity from cartridge (A_{eluted}) (mCi)[a]	Residual activity on cartridge after elution ($A_{C\_residual}$) (mCi)[a]	Trapping efficiency (%)[b]	Elution efficiency (%)[c]
5.64	1.66	0.28	3.55	0.16	66	96
5.54	1.63	0.03	3.85	0.06	70	99
5.62	1.65	0.03	3.91	0.09	70	99
5.57	1.48	0.06	3.99	0.08	72	99

[a]All measurements are decay corrected. [b]Trapping efficiency (%) was calculated as $A_{C\_trapped} / A_0$, where $A_{C\_trapped} = A_0 - (A_{waste} + A_{0\_residual})$ is the amount trapped on the cartridge at the end of the trapping step. [c]Elution efficiency (%) was calculated as $A_{eluted} / A_{C\_trapped}$.

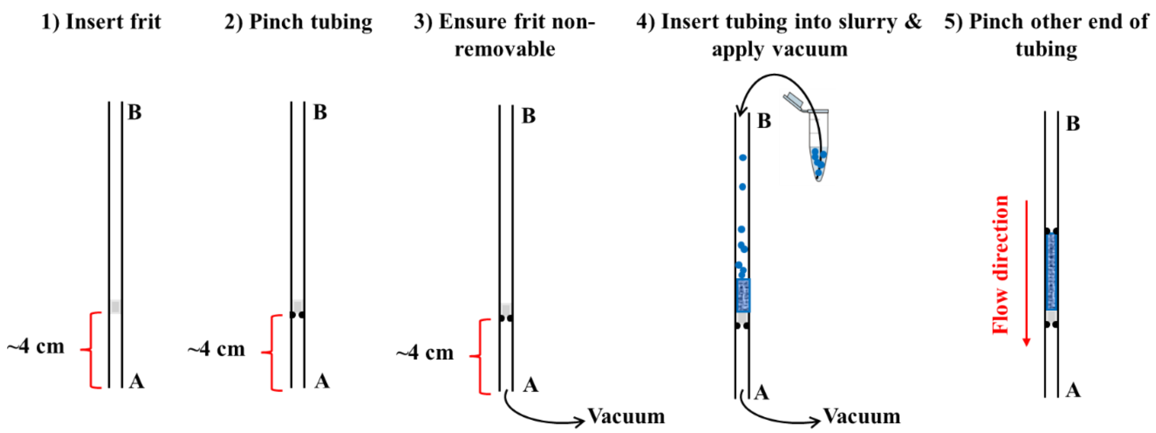

Figure S 16. Preparation of micro cartridge for the purification of [^{18}F]FVSB

4.5 Analysis of ^{18}F-labeled RGDC peptide

A. Crude ^{18}F-labeled RGDC peptide

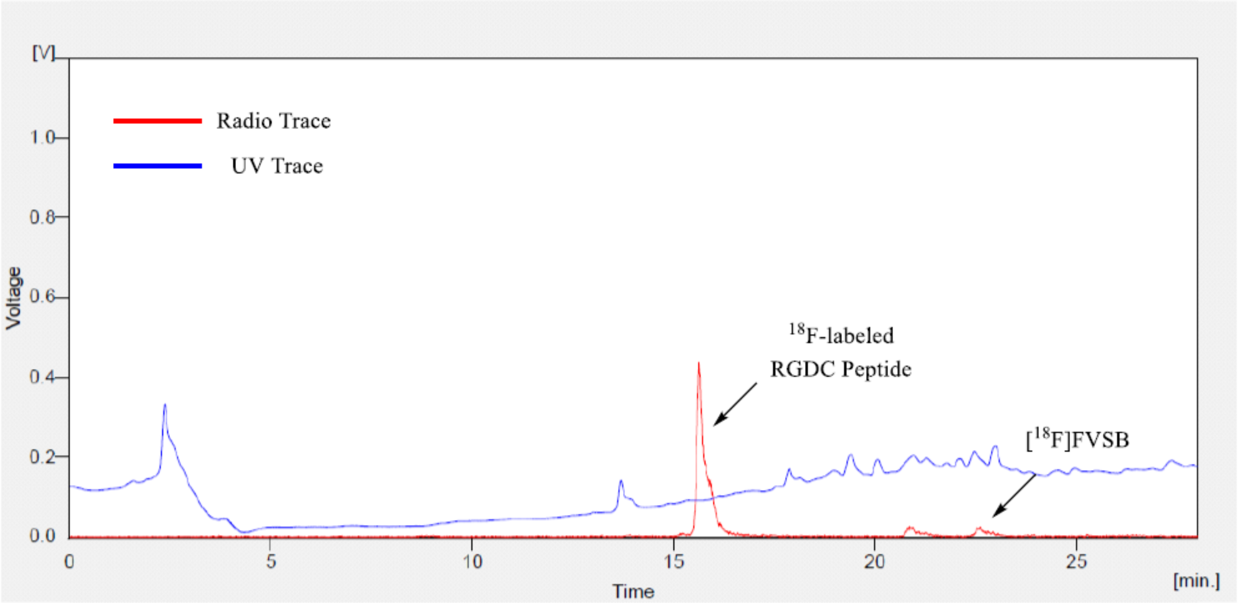

Figure S 17. Example of analytical HPLC chromatogram for crude ^{18}F-labeled RGDC peptide produced using the optimized microscale synthesis.

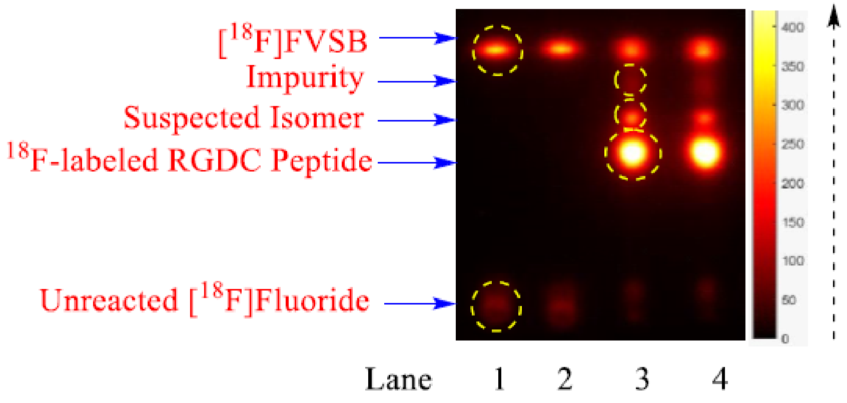

Figure S 18. TLC analysis of crude [^{18}F]FVSB and crude ^{18}F-labeled RGDC peptide. A sample of crude [^{18}F]FVSB was spotted in lanes 1 and 2, and a sample of the crude ^{18}F-labeled RGDC peptide was spotted in lanes 3 and 4. Dashed arrow represents propagation of mobile phase.

In lanes 3 and 4 of the radio-TLC image shown in **Figure S18,** there is a small band slightly overlapping the band corresponding to the radiotracer (^{18}F-labeled RGDC). However, in the HPLC chromatogram of the same sample in **Figure S17,** the crude ^{18}F-labeled RGDC peptide contains a single radioactive peak. (There may be a small shoulder on the main peak but it is not resolved.) We hypothesize there may be a small amount of an isomer present but since they have not been resolved in prior work, we include both bands as the product band in our TLC analyses, to enable comparison of the reaction performance in this work to prior reports.

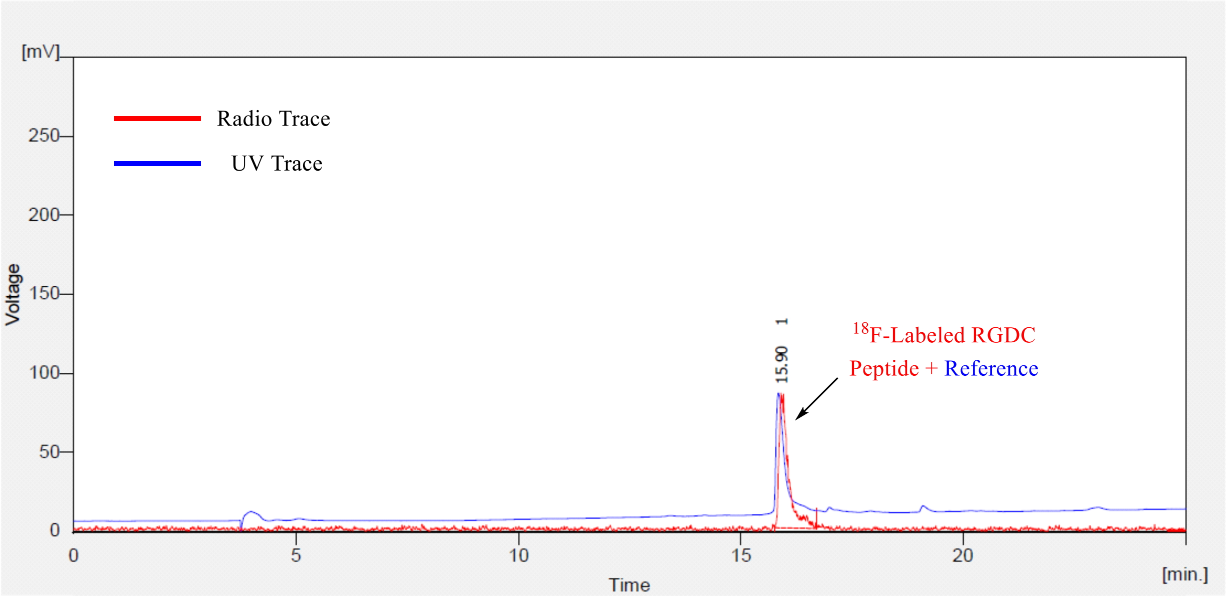

Figure S 19. Example analytical HPLC chromatogram for a co-injection of HPLC-purified ^{18}F-labeled RGDC peptide with the reference standard.

5. Molar Activity

To enable determination of molar activity of [^{18}F]FVSB, a calibration curve was generated **(Figure S20**). Different molar amounts of FVSB reference standard were injected into HPLC and, for each injection, the area under the curve for the peak in the resulting UV absorbance chromatogram was computed and plotted against the amount of FVSB. Using a linear fit and uncertainties the in parameters, the LOD was determined to be 0.62 nmol.

To determine the molar activity of a sample of [^{18}F]FVSB, we first purified it via analytical HPLC using the same conditions as described in Materials and Methods, section 4.5. The purified [^{18}F]FVSB was then concentrated by trapping on a micro-cartridge and eluting with 140 µL of MeOH (Supplementary Materials, Section 4.4) and then re-injected in HPLC for molar activity analysis (**Figure S21**). The injected radioactivity was 745 µCi, and since no UV absorbance peak was visible, we estimate the molar activity using the LOD to be > 45 GBq/µmol.

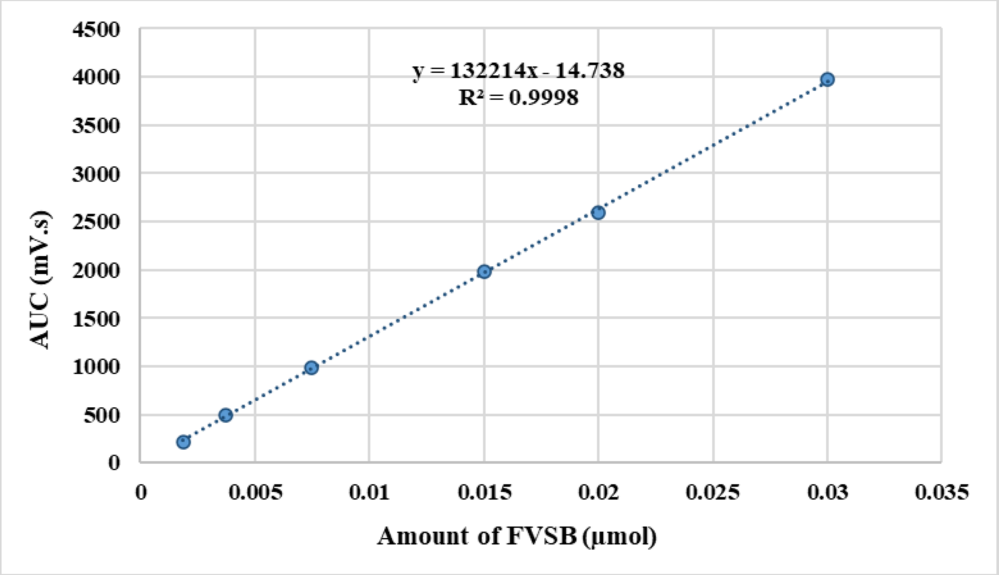

Figure S 20. Calibration curve for molar activity determination of [^{18}F]FVSB. AUC = Area under the curve of the UV absorbance peak corresponding to FVSB.

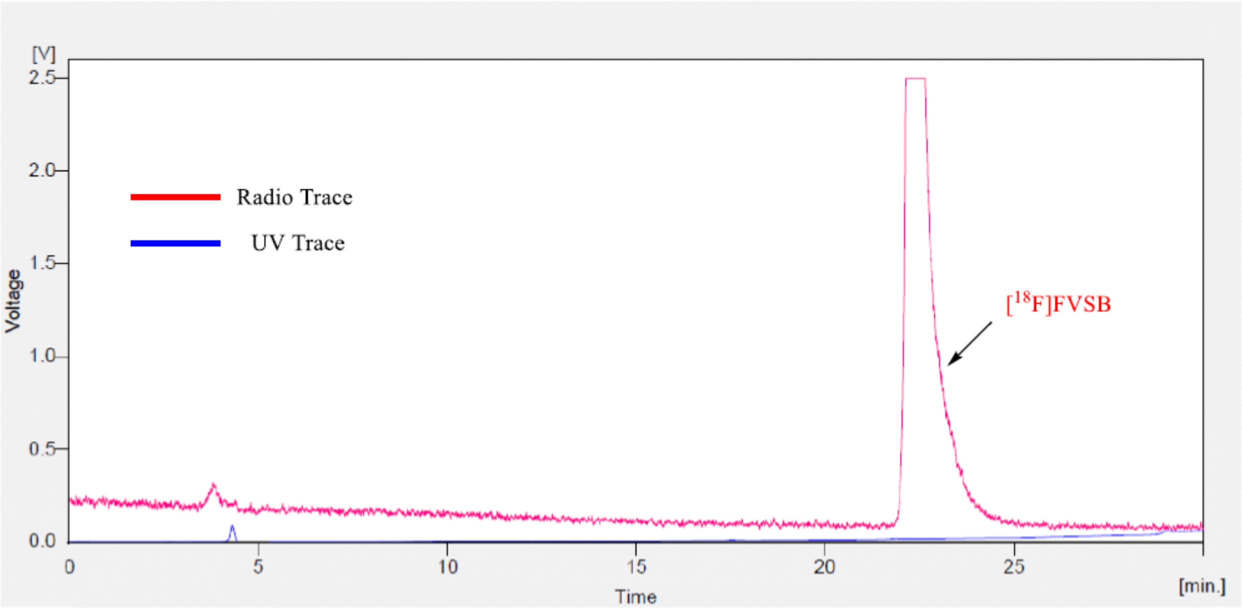

Figure S 21. Example analytical HPLC chromatogram of HPLC-purified [^{18}F]FVSB for molar activity determination.

To enable determination of molar activity of the ^{18}F-labeled peptide, another calibration curve was generated using the same approach (**Figure S22**). After purification of the ^{18}F-labeled peptide via analytical HPLC, a 60 µL aliquot of the HPLC-purified ^{18}F-labeled peptide was injected into the analytical HPLC system for determination of molar activity (**Figure S23**). The injected radioactivity was measured to be 75 µCi, and based on the area under the curve of the UV absorbance peak, the amount of mass was estimated to be 3.1 nmol. In a later follow-up study, we found there may be a co-eluting impurity (i.e. an FVSB-precursor-derived impurity conjugated with the peptide). Thus, we are able to estimate the apparent molar activity as 0.588 GBq/µmol.

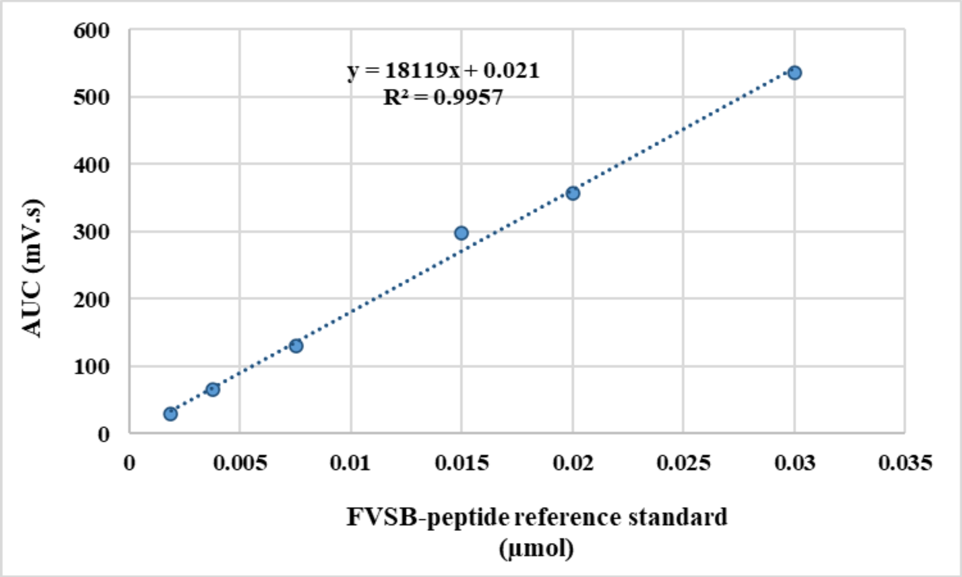

Figure S 22. Calibration curve for molar activity determination of ^{18}F-labeled RGDC peptide. AUC = Area under the curve of the UV absorbance peak corresponding to FVSB-peptide reference standard.

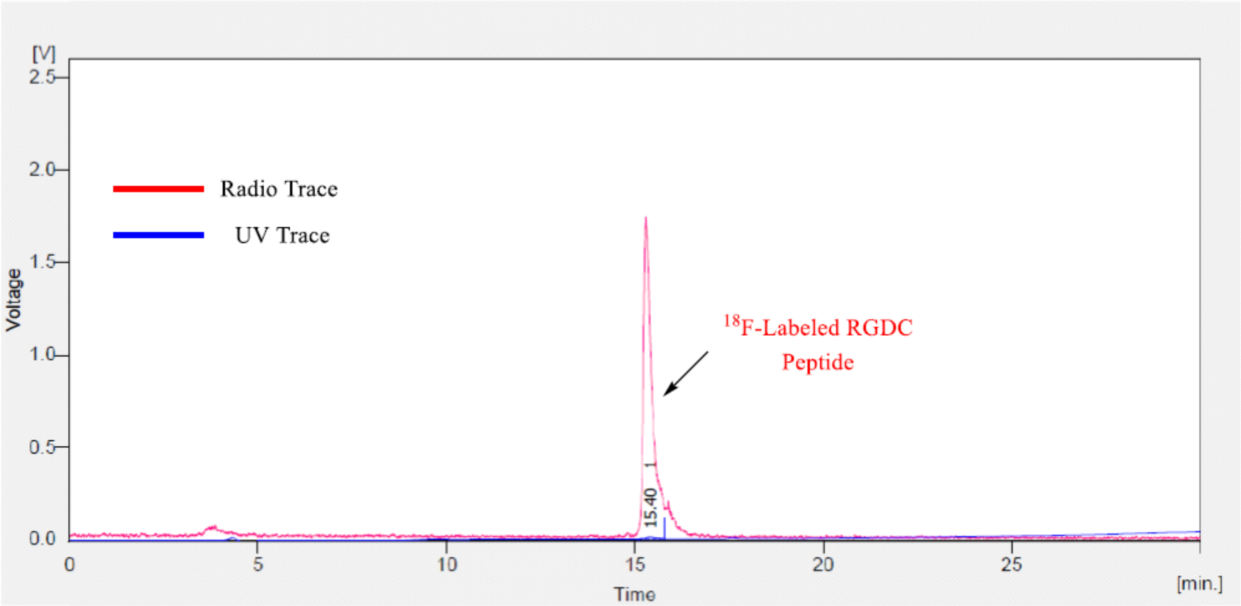

Figure S 23. Example analytical HPLC chromatogram of HPLC-purified ^{18}F-labeled RGDC peptide for determination of molar activity.

6. NMR spectra of synthesized compounds

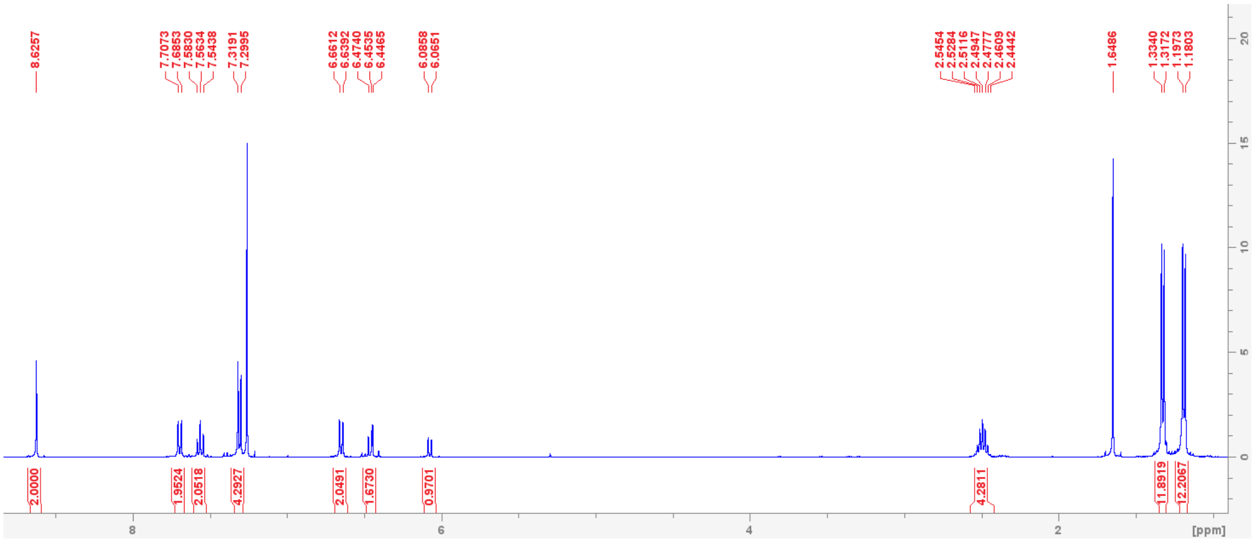

Figure S 24. ^{1}H NMR spectrum of FVSB precursor (**7**) in $CDCl_3$. **^{1}H NMR** (400 MHz, $CDCl_3$, δ): 8.63 (s, 2H), 7.70 (d, *J* = 9.0 Hz, 2H), 7.56 (t, *J* = 7.8 Hz, 2H), 7.31 (d, *J* = 7.9 Hz, 4H), 6.65 (d, *J* = 8.8 Hz, 2H), 6.47-6.45 (m, 2H), 6.08 (d, *J* = 8.3 Hz, 1H), 2.49 (hept, *J* = 6.9 Hz, 4H), 1.33 (d, *J* = 6.7 Hz, 12H), 1.18 (d, *J* = 6.8 Hz, 12H).

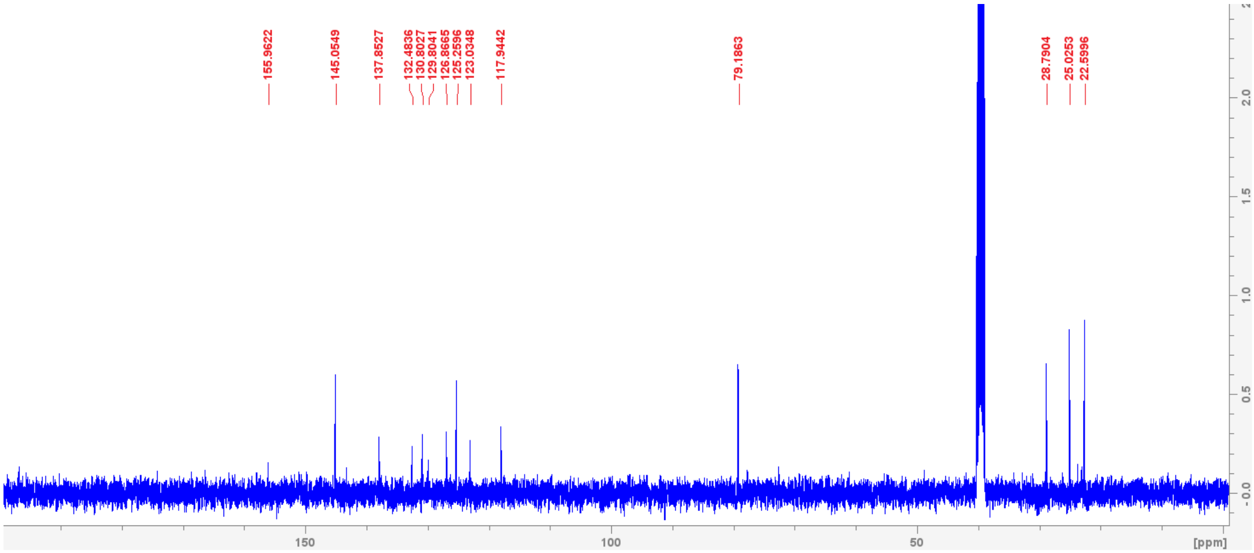

Figure S 25. ^{13}C NMR spectrum of FVSB precursor (7) in DMSO. **^{13}C NMR** (100 MHz, DMSO, δ): 155.9, 145.1, 137.9, 132.5, 130.8, 129.8, 126.9, 125.3, 123.0, 117.9, 79.2, 28.8, 25.0, 22.6.

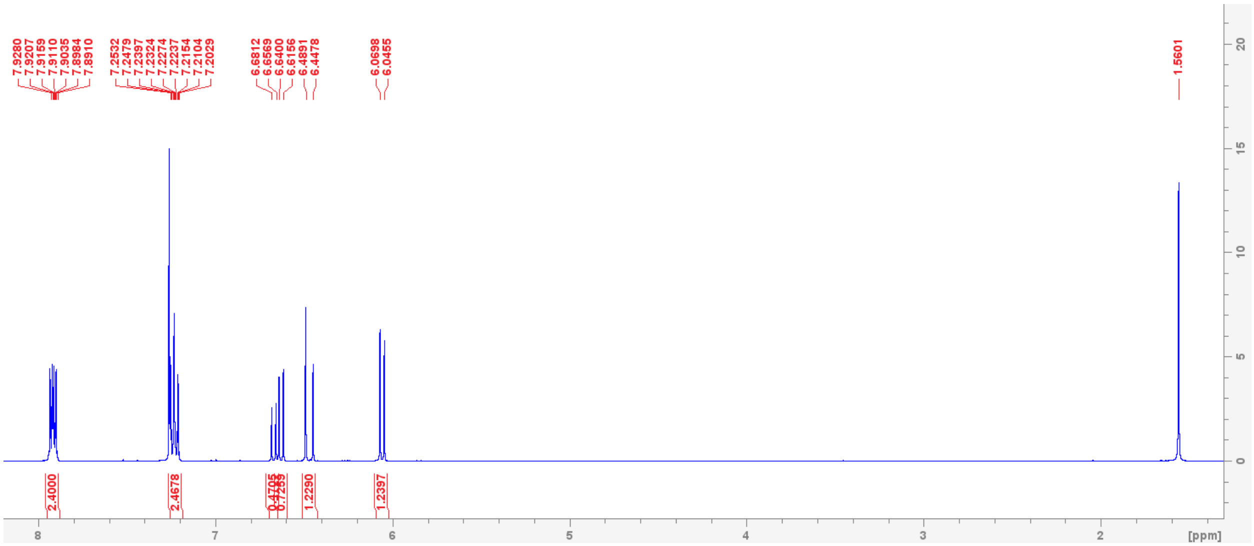

Figure S 26. ^{1}H NMR spectrum of FVSB reference standard in $CDCl_3$. **^{1}H NMR** (400 MHz, $CDCl_3$, δ): 7.94-7.89 (m, 2H), 7.25-7.20 (m, 2H), 6.67 (dd, *J* = 9.8, 1H), 6.62 (d, *J* = 9.7 Hz, 1H), 6.46 (d, *J* = 16.5 Hz, 1H), 6.06 (d, *J* = 9.7 Hz, 1H).

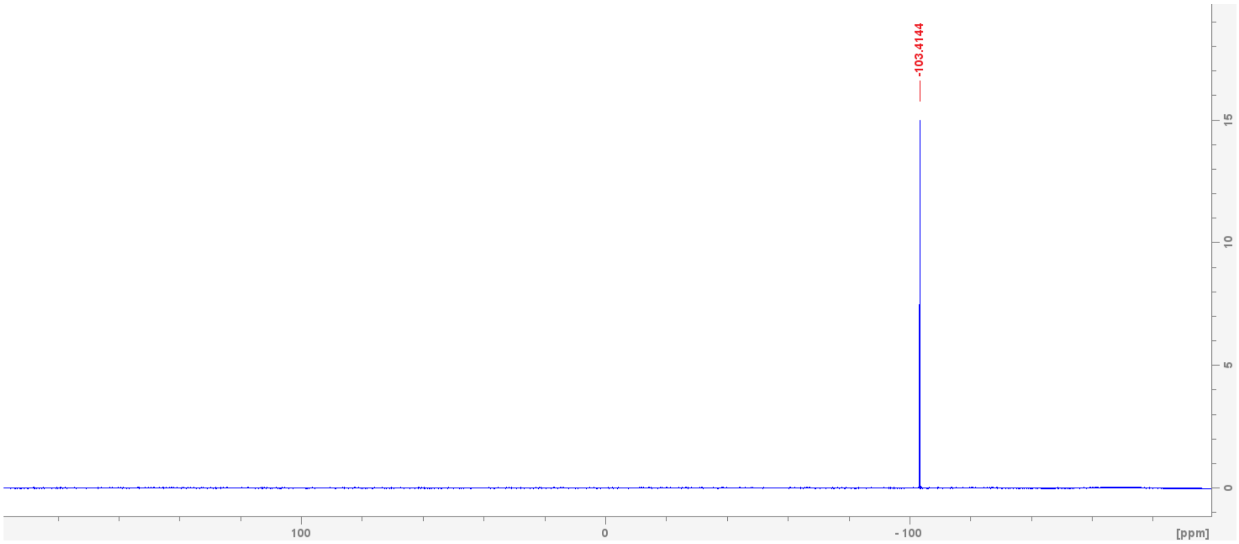

Figure S 27. ^{19}F NMR spectrum of FVSB reference standard in $CDCl_3$. **^{19}F NMR** (376 MHz, $CDCl_3$, δ): -103.4.

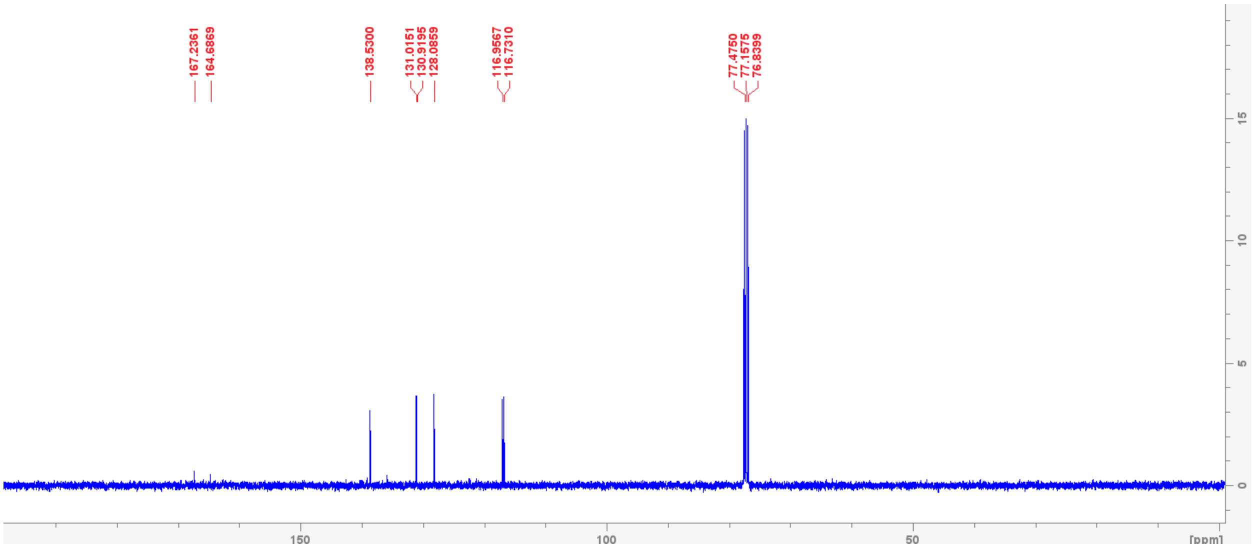

Figure S 28. ^{13}C NMR spectrum of FVSB reference standard in $CDCl_3$. **^{13}C NMR** (100 MHz, $CDCl_3$, δ): 167.2, 164.7, 138.5, 131.5 (d, J_{FC} =9.6 Hz), 128.1, 116.8 (d, J_{FC} = 22.4 Hz).

7. References

1. Lu, Y.; Chao, P.H.; Collins, J.; van Dam, R.M. Rapid Concentration of Ga-68 and Proof-of-Concept Microscale Labeling of [68Ga]Ga-PSMA-11 in a Droplet Reactor. *Molecules* **2024**, *29*, 4572, doi:10.3390/molecules29194572.
